# Supplementary material for: Cohort profile: the Dutch famine birth cohort (DFBC)— a prospective birth cohort study in the Netherlands
Source: BMJ Open. 2021 Mar 4;11(3):e042078. doi: 10.1136/bmjopen-2020-042078 (PMC7934722; doi:10.1136/bmjopen-2020-042078)
Supplement: Author's manuscript [file bmjopen-2020-042078.draft_revisions.pdf]

BMJ Open is committed to open peer review. As part of this commitment we make the peer review history of every article we publish publicly available.

When an article is published we post the peer reviewers' comments and the authors' responses online. We also post the versions of the paper that were used during peer review. These are the versions that the peer review comments apply to.

The versions of the paper that follow are the versions that were submitted during the peer review process. They are not the versions of record or the final published versions. They should not be cited or distributed as the published version of this manuscript.

BMJ Open is an open access journal and the full, final, typeset and author-corrected version of record of the manuscript is available on our site with no access controls, subscription charges or pay-per-view fees (http://bmjopen.bmj.com).

If you have any questions on BMJ Open's open peer review process please email info.bmjopen@bmj.com

BMJ Open

Cohort Profile: The Dutch famine birth cohort (DFBC), a prospective birth cohort study in the Netherlands

Journal:	*BMJ Open*
Manuscript ID	bmjopen-2020-042078
Article Type:	Cohort profile
Date Submitted by the Author:	24-Jun-2020

Complete List of Authors:	Bleker, Laura ; Amsterdam UMC Location AMC, Epidemiology and Data Science<br>de Rooij, Susanne; Amsterdam UMC Location AMC, Epidemiology and Data Science<br>Painter, Rebecca; Amsterdam UMC Location AMC, Obstetrics & Gynaecology<br>Ravelli, Anita C.J.; Amsterdam UMC Location AMC, Medical Informatics<br>Roseboom, Tessa; Amsterdam UMC Location AMC, Clinical Epidemiology and Biostatistics; Obstetrics and Gynaecology

Keywords:	EPIDEMIOLOGY, NUTRITION & DIETETICS, PUBLIC HEALTH

SCHOLARONE™ Manuscripts

I, the Submitting Author has the right to grant and does grant on behalf of all authors of the Work (as defined in the below author licence), an exclusive licence and/or a non-exclusive licence for contributions from authors who are: i) UK Crown employees; ii) where BMJ has agreed a CC-BY licence shall apply, and/or iii) in accordance with the terms applicable for US Federal Government officers or employees acting as part of their official duties; on a worldwide, perpetual, irrevocable, royalty-free basis to BMJ Publishing Group Ltd ("BMJ") its licensees and where the relevant Journal is co-owned by BMJ to the co-owners of the Journal, to publish the Work in this journal and any other BMJ products and to exploit all rights, as set out in our licence.

The Submitting Author accepts and understands that any supply made under these terms is made by BMJ to the Submitting Author unless you are acting as an employee on behalf of your employer or a postgraduate student of an affiliated institution which is paying any applicable article publishing charge ("APC") for Open Access articles. Where the Submitting Author wishes to make the Work available on an Open Access basis (and intends to pay the relevant APC), the terms of reuse of such Open Access shall be governed by a Creative Commons licence – details of these licences and which Creative Commons licence will apply to this Work are set out in our licence referred to above.

Other than as permitted in any relevant BMJ Author's Self Archiving Policies, I confirm this Work has not been accepted for publication elsewhere, is not being considered for publication elsewhere and does not duplicate material already published. I confirm all authors consent to publication of this Work and authorise the granting of this licence.

For peer review only

Cohort Profile: The Dutch famine birth cohort (DFBC), a prospective birth cohort study in the Netherlands

Laura S. Bleker[1], Susanne R. de Rooij[1*], Rebecca C. Painter[2], Anita C.J. Ravelli[2-3], Tessa J. Roseboom[1-2]

[1]Department of Epidemiology and Data Science, Amsterdam UMC, University of Amsterdam, The Netherlands

[2]Department of Obstetrics and Gynecology, Amsterdam UMC, University of Amsterdam, The Netherlands

[3]Department of Medical Informatics, Amsterdam UMC, University of Amsterdam, The Netherlands

*Corresponding author:

Dep. Epidemiology and Data Science, Amsterdam UMC, University of Amsterdam

Meibergdreef 9, 1105 AZ Amsterdam, The Netherlands

Email: s. r.derooij@amsterdamumc.nl

Tel nr: **31 20 56 64660

Word count: 4297

Abstract

Purpose: The Dutch famine birth cohort study is a quasi-experimental cohort study which was set up to investigate the effects of acute maternal undernutrition during specific stages of gestation on the offspring's adult health, with a particular focus on chronic cardiovascular and metabolic diseases, ageing and mental health.

Participants: Cohort members include 2414 singletons born alive and at term in the Wilhelmina Gasthuis in Amsterdam around the time of the Dutch famine (1943-1947).

Findings to date: Since 1994, several rounds of interviews, physical examinations and data linkage have been collected, allowing repeated measures of a wide range of phenotypic information, including medical history, signs and symptoms of cardiovascular disease, mental health and quality of life, as well as functional tests of the heart, lungs and kidneys. Biological samples have been collected repeatedly (blood, urine and buccal swabs) and lipid and glucose profiles have been assessed, as well as HPA-axis functioning, autonomic nervous system activity, genetic and epigenetic measurements. Also, medical imaging has been used to assess structure and function of carotid and femoral arteries as well as brain structure and function. Studies have shown that prenatal famine exposure has lasting effects on health in later life with robust evidence for associations with cardio-metabolic and mental health. The effects were dependent of its timing during gestation (and the organs and tissues growing at that time), with most effects observed for those exposed to the famine during early gestation. The effects were independent of size at birth.

Future plans: As the cohort ages, we will be assessing the effects of prenatal undernutrition on (brain) ageing, cognitive decline and dementia. Plans also include studying cardio-metabolic and mental health and mortality up until around 76 years of age.

For peer review only

Strengths and Limitations: Strengths of the study include its quasi-experimental design and the long period of follow up. A limitation is a limited power to show effects of prenatal undernutrition on adult outcomes especially for those that are relatively rare. In addition, selective participation and selective survival may have occurred.

Registration: The Dutch famine birth cohort is not linked to a clinical trial.

Keywords : Dutch famine, undernutrition, pregnancy, fetal programming, cardiovascular disease, metabolic disease, brain, ageing

For peer review only

Introduction

Study rationale

The Dutch famine birth cohort study is a birth cohort of men and women born around the time of the 1944-1945 Dutch famine in the Wilhelmina Gasthuis in Amsterdam, the Netherlands. The study was set up to investigate the effects of prenatal exposure to the Dutch famine on adult health.

Fetal origins of health and disease

Epidemiological studies in populations across the globe have consistently shown that birth weight is inversely associated with levels of blood pressure , serum cholesterol, type 2 diabetes and cardiovascular morbidity and mortality in adulthood (1-7). In 1986, Barker & Osmond suggested that undernutrition during gestation could hamper growth in utero and lead to small size at birth as well as an increased risk of disease in later life (8). In experimental studies in animals, undernutrition during pregnancy induced cardiovascular and metabolic changes in the adult offspring (9, 10). These studies suggest that poor fetal nutrition has long-lasting consequences for adult health. The "Fetal Origins of Health and Disease" hypothesis by David Barker proposed that environmental stimuli during fetal life induce 'programming' – a term referring to permanent structural, physiological and metabolic changes in the fetus. While these changes could infer certain fetal advantages in the short term, they were hypothesized to predispose to susceptibility to cardiovascular and metabolic dysfunction and subsequent disease in adult life (11). Early prenatal life in particular is considered a 'critical' period, a period in which the fetus is especially susceptible for external influences, since organ structures and associated functions undergo rapid development during that time, and the set-point of physiological and metabolic responses is determined (12). Insufficient nutrition during prenatal

development may thus permanently alter certain structural and physiological functions of the embryo or fetus, eventually leading to poorer health in adulthood. However, human observational studies are limited by the fact that birth weight is affected by many factors besides maternal nutrition and does not always adequately reflect nutritional status of the mother during her pregnancy (13). To bypass potential confounding effects of socio-environmental and genetic factors on birth weight, semi experimental studies can offer an elegant solution to investigate causality of short and long-term effects of prenatal undernutrition in humans.

The Dutch famine birth cohort study is the first study in humans to directly investigate the long-term effects of prenatal undernutrition during different trimesters of gestation on adult physical and mental health. During World War II, part of the Dutch population was exposed to famine during the severe 'hunger winter' of 1944-1945. This historical tragedy has provided the unique opportunity to study short and long-term effects of famine exposure during intrauterine development. The Dutch famine can be viewed as a 'natural experiment', because of its sudden onset and end in a previously well-nourished population. The famine was acute, relatively short, and had an impact on the entire population within the affected regions, minimizing confounding by genetic inheritance or other simultaneously occurring risk factors, such as socio-economic status. Also, throughout the famine food rations were precisely registered. It is therefore not surprising that many researchers have been interested in studying individuals prenatally exposed to the Dutch famine, from birth up to adulthood, using various methodological constructs.

Historical events leading to the Dutch famine

In 1944-1945, the Dutch famine hit the western parts of the Netherlands towards the end of World War II, only months before its liberation from the German occupier by the Allied forces. Before the war, nutritional standards of the Dutch population were generally adequate (14). On

the 10th of May 1940, the Netherlands were invaded by German forces, with immediate impact on the Dutch food supply. Food imports from other countries, including the Dutch colonies, were no longer possible, and part of the food produced in the Netherlands was sent to Germany (14). The National Bureau for Food Distribution had already implemented food rationing and distribution prior to the invasion, and was fully operative throughout the entire country by the end of April 1941. The food rations were determined weekly and recorded in detail. However, daily rations remained calorically and nutritionally adequate until September 1944, when they suddenly dropped below 1600 calories (15, 16). On the 17th of September 1944, the Dutch exiled government requested a railroad strike to support the advance of the Allied forces, which by that time had already liberated the southern part of the Netherlands. The German occupier responded by an abrupt ban of food transports to the western part of the Netherlands. By November 1944, food stocks in the large cities in the west of the Netherlands had been exhausted, and the ban was partly lifted to allow food transport across water. However, due to an early and exceptionally harsh winter, waterways and canals had frozen over. Food stocks could no longer be replenished and famine set in for the inhabitants of the large cities in the west, including Amsterdam, Rotterdam and The Hague. The official daily rations per person dropped to below 1000 calories in November 1944 and varied between 400 and 800 calories in the following six months, a period that would later be referred to as the "Hunger Winter". Children aged 1 year or younger were relatively protected, as their official daily ration never dropped below 1000 calories. Although this initially also applied to pregnant and lactating women, this was no longer possible as the food scarcity persisted. On the fifth of May 1945, the western parts of the Netherlands were liberated by The Allied Forces. Within a week, daily rations had rapidly risen above 1000 calories, through supplements by airdrops and the Special Red Cross Feeding Team (17). By June 1945, rations had risen above 2000 calories. Within weeks, the

For peer review only

pre-famine nutritional standards were re-established, and were maintained until the food ration was permanently lifted, 5 years after the war in 1950 (18, 19).

Consequences of prenatal undernutrition

Some of the earliest studies on the Dutch famine were published within a few years after World War II by Smith and Sindram, and focused on the effects of prenatal famine exposure on birth outcomes. Based on the assumption of a severe generalized undernutrition in urban areas, they obtained birth records from babies born around the Dutch famine in hospitals in Amsterdam, Rotterdam, and The Hague, and compared their birth weights to that of babies born before the famine. Birth weight was shown to be 240 to 340 grams lower in those exposed to the Dutch famine in utero (20, 21).

In the 70's, Professor Zena Stein and Professor Mervin Susser were primarily interested in the effects of prenatal undernutrition on mental performance in early adulthood. They used data from 19-year old Dutch military conscripts, who had all been medically examined and psychologically tested. Given their date and place of birth, Stein and Susser retrospectively reconstructed birth cohorts prenatally exposed to famine (defined as born in one of seven famine-stricken cities in the west: Amsterdam, Leiden, Haarlem, Utrecht, the Hague, Rotterdam, Delft) and compared them to cohorts of individuals who had not been exposed (defined as born in one of the control cities in the north, east or south: Maastricht, Heerlen, Breda, Tilburg, Eindhoven, Enschede, Helmond, Hengelo, Zwolle, Leeuwarden, Groningen). They did not find an association between the frequency of a diagnosis of 'severe mental retardation' or Intelligence Quotient and prenatal famine exposure in 19-year old men (19). However, an increased prevalence of obesity was shown among those military conscripts who were exposed to famine during early gestation, a relatively rare condition at the time (22, 23).

Thereafter, in 1975, Stein and Susser replicated findings from Smith and Sindram using data from additional famine-stricken cities. They collected birth records of maternities from teaching hospitals in affected areas (Amsterdam, Leiden, and Rotterdam) and in non-affected areas (Groningen in the north, Heerlen in the south). In their analyses, they compared birth cohorts defining exposure both according to timing of birth (born outside or during the famine, in the same area) and on place of birth (born in affected or unaffected areas, in the same period). They showed that food intake in the third trimester negatively affected birth weight, infant length, head circumference, placental weight and maternal weight at the end of pregnancy. Moreover, the effects on size at birth were shown to be independent of gestational age and occurred only below an empirical threshold of an average daily food ration of 1500 calories or less (24, 25).

Another approach twenty years later by Ezra Susser, Hoek and Brown comprised of retrospectively analysing data from the Dutch Psychiatry Registry. The registry ascertains cases of psychiatric disorder resulting in hospitalization, and included information on diagnosis as well as on date and place of birth. Based on this information, cases were retrospectively allocated to exposed or unexposed birth cohorts. Increased rates for schizophrenia (26, 27), and affective disorders (28, 29) were observed in those prenatally exposed to famine. Re-entering the data from military conscripts in men, higher rates for schizophrenia (30), a schizoid personality (31) and an antisocial personality disorder (32), based on the psychiatric assessments at conscription using the International Classification of Diseases, were described in individuals exposed to the Dutch famine during intrauterine development.

A retrospective female Dutch famine birth cohort was established during the 1980-1990's, prospectively examining next generation reproductive outcomes after maternal prenatal famine exposure, including their offspring's birth weight (33, 34). All 1116 women born during the famine in the Wilhelmina Gasthuis (WG) in Amsterdam and before and after the famine (Aug

1, 1944- April 15, 1946) were traced through the national population registers. Information on their date of birth and birthweight was collected from medical records retrieved from the city archives of Amsterdam. A total of 683 women were interviewed at their homes at the mean age of 43 years, and were all able to recall the birth weight of their children (34). This study design avoided sampling bias as a specific set of adult outcomes was assessed in an entire birth cohort of exposed female individuals. The authors concluded that maternal prenatal famine exposure did not affect the association between maternal and offspring birth weight.

For peer review only

Cohort description

Eligibility

In 1994, the Dutch famine birth cohort was set up in the Academic Medical Centre in Amsterdam, the Netherlands. All men and women born as term singletons between November 1, 1943 and February 28, 1947 in the Wilhelmina Gasthuis (WG) in Amsterdam were eligible candidates to be included in the Dutch famine birth cohort. The WG was the main maternity hospital in Amsterdam at the time of the famine and birth records were still kept at the city archive of Amsterdam (34). Only live born singletons after a full-term pregnancy were included (≥259 days gestational age, calculated either from the date of the last menstrual period or by the obstetrician's estimation at first prenatal visit and at physical examination of the child at birth).

Study participants

The birth ledgers, which contained general information on date of birth, name of the parents and identification number were retrieved from the Obstetrics department of the Academic Medical Center (the former WG) in Amsterdam of individuals born between November 1, 1943 and February 28, 1947. From the 5425 records, all individuals exposed to famine, according to our definition (see below), were considered eligible exposed candidates. A random sample of eligible individuals unexposed to famine during gestation (born before the famine or conceived after the famine) was taken. Twins and stillbirths were excluded. This resulted in 2680 eligible candidates for which the detailed medical records were searched and coded in all detail in the city archive of Amsterdam. Of these 2680 individuals, 27 individuals (1.0%) were excluded because their medical records were missing or incomplete, and 239 individuals (8.9%) were excluded based on their preterm birth (<259 days gestational age). Ultimately, 2414 people

were considered eligible (821 live born term singletons exposed to famine prenatally, 764 live born term singletons born before the famine and 829 live born term singletons conceived after the famine).

Exposure to famine

Exposure to famine during gestation was defined as an average maternal daily ration of less than 1000 calories during any 13-week period of gestation (35). According to the official daily rations of the general population, children born in Amsterdam between January 7, 1945 and December 8, 1945, were considered to be exposed to famine during prenatal life. Three 16-week periods were distinguished; children who were mainly exposed during ***late*** gestation (born between January 7 and April 28, 1945), ***mid*** gestation (born between April 29 and August 18, 1945) or ***early*** gestation (born between August 19 and December 8, 1945). A sample of the individuals born within one year before the famine or conceived up to one year after the end of the famine were eligible for controls as comparisons. Being unexposed to famine was defined as being born before the famine (born between November 1, 1943 and January 7, 1945), or conceived after the famine (born between December 9, 1945 and February 28, 1947) (Figure 1).

Figure 1. Dutch famine birth cohort schematic presentation of gestational famine exposure.

Maternal and birth characteristics

Maternal and birth characteristics of the 2414 eligible men and women are presented in Table 1, according to timing of exposure to famine (born before the famine, exposed in late gestation, exposed in mid-gestation, exposed in early gestation, conceived after the famine). Medical

For peer review only

records contained information on maternal characteristics including maternal weight and blood pressure throughout pregnancy as well as a detailed description of delivery and the postpartum period. Also, they included detailed information about the size of the baby and placenta at birth. Records were completed at every prenatal visit (usually every 2 weeks), typically starting at the end of the second trimester.

Maternal characteristics included maternal age, marital status, reproductive and medical history, and occupation of the head of the family. Blood pressure and maternal weight were measured. Pelvic measurements were taken, including the interspinous distance (the distance between the anterior superior iliac spines). Maternal weight gain in the third trimester was estimated as the difference between the woman's weight at the last prenatal visit and her weight as close as possible to the start of the third trimester, multiplied by the ratio of trimester duration (13 weeks) and the time interval between the two weight measurements.

Birth characteristics included date of birth, sex, birth weight, and birth length (crown-to-heel). Head circumference was calculated by: π * (biparietal diameter + occipitofrontal diameter) * 0.5. Ponderal index was calculated by: birth weight / (crown-to-heel^2). Placental area was calculated by: π * placental length * placental width * 0.25. At discharge, infant weight and type of feeding (exclusive breastfeeding/exclusive bottle-feeding/a combination) were recorded. Besides information from the medical records for each women the profession of the father / the head of the family was collected using the hospital admission "crib cards" and coded as manual or non-manual work. In 1945, 57% of the population in Amsterdam was estimated to be of poor social economic class. The WG hospital typically facilitated deliveries for unmarried women and women with poor housing/ socioeconomic conditions [35].

For peer review only

Table 1. Maternal characteristics and birth outcomes of 2414 singleton children born alive and at term in the Wilhelmina Gasthuis in Amsterdam between 1 November 1943 and 28 February 1947.

		**Exposure to Famine**				**Total (SD)**	**Missing (*n*)**
	**Born before n=764**	**Late gestation n=307**	**Mid gestation n=297**	**Early gestation n=217**	**Conceived after n=829**	**n=2414**	
**Maternal characteristics**							
Age (years)	28.5	30.0	28.1	27.7	28.0	28.3 (6.4)	0
Never married (%)	13.2	9.8	20.2	25.8	16.3	15.8	0
Weight gain third trim. (kg)	3.1	0.0	4.9	5.7	4.2	3.5 (3.2)	732
Weight at last prenatal visit (kg)	66.7	61.8	63.5	67.9	69.1	66.6 (8.7)	281
Interspinous distance (cm)	26.1	25.8	25.5	25.8	26.3	26.0 (1.8)	14
**Birth outcomes**							
	**Born before *n*=764**	**Late gestation *n*=307**	**Mid gestation *n*=297**	**Early gestation *n*=217**	**Conceived after =829**	**Total *n*=2414**	
Boys (%)	53.1	48.2	48.5	49.8	52.5	51.4	
First born (%)	39.7	29.6	36.7	39.2	39.3	37.9	
Pregnancy duration (days)	285	283	285	287	286	285 (11)	371

Birth weight (g)	3373	3133	3217	3470	3413	3346 (478)	0
Body length (cm)	50.5	49.4	49.8	50.9	50.5	50.3 (2.2)	32
Ponderal index (kg/m^3)	26.1	25.8	26.0	26.2	26.5	26.4 (2.4)	32
Head circumference (cm)	32.9	32.3	32.1	32.8	33.2	32.8 (2.4)	17
Placental diameter (cm)	20.6	19.7	20.0	19.9	20.2	20.2 (2.5)	357

*Adapted from Table 1, Page 59, Ravelli et al. (36)

Follow-up

In 1994, current addresses of the children born before, during and after the famine were requested from the population registry (Bevolkingsregister) in Amsterdam, which traced 2155 of the 2414 eligible participants. Of these, 265 persons had died, 199 had emigrated, and another 164 refused to share their address, resulting in 1527 (63.3%) of 2155 cases. Power calculations for a study sample to be invited for interviewing and clinical measurements were based on the 120-min glucose concentrations after a 75 gram oral glucose load as the main outcome variable, based on pooled data from three previous MRC Southampton studies (Hertfordshire, Preston and Sheffield). To detect an increase of 10% in the 120-minute glucose concentration, a sample size of 700 men and women (450 prenatally exposed and 250 unexposed persons) was needed for 93% power and a 5% type 1 error probability (two-sided). A 70% participation rate for the oral glucose load test was assumed and based on this, interviews with 1000 men and women at their homes were planned. We invited all people in each of the 5 exposure/non-exposure groups, starting with those who lived in, or close to, Amsterdam (n=1018) to participate in the first

study, of whom 912 (90%) agreed to be interviewed at their homes and 741 (81%) of the interviewed participants visited the clinic for measurements (35).

Data collection of the Dutch famine birth cohort study has occurred in five study waves from 1994/6 until 2018, and a sixth wave is currently (2019-2020) in progress. Measurements consisted of a home interview at age 50 and a hospital visit around the ages of 50 (wave I, 1994 - 1996), 58 (wave II, 2002-2004), 63 (wave III, 2008-2009) and 68 (wave IV, 2013), and completion of a questionnaire by the participants at age 73 (wave V, 2018). Another hospital visit at age 74-75 is currently ongoing (wave VI, 2019-2020). At wave V in 2018, 1207 of the 1527 participants were still living in the Netherlands with known addresses (79%), of which 595 agreed to participate (49%). The sixth wave is currently in progress.

Measurements

Table 2 gives a summary of the study waves, including the mean ages of the famine exposed participants during that wave, with outcome measures and a description of participation rates.

Patient and Public Involvement statement

Cohort members are informed about study aims and outcomes via newsletters and the studies website www.hongerwinter.nl. Cohort members have not been involved in setting up research questions and data collection, study design, recruitment strategies, assessment of study burden and dissemination of study results.

only

Table 2. Dutch famine birth cohort study waves, including the mean ages of the participants at examination, the outcome measures and a description of attrition rates.

Phase	Measurement	Loss of follow-up
**Wave I**<br><br>1994-1996	**Wave 1a birth data collection/ exposure definition/ mortality status**	
**Wave I**<br><br>(Age 50)<br><br>1994– 1996	**Wave Ib (home interview)**<br><br>- Questionnaires (General information, medical information, life style factors, physical activity, weight history, reproductive history, EPIC Food frequency questionnaire, self-perceived health and medication use) (37, 38)<br>- Anthropometrics (39)<br>- Blood pressure (40, 41)<br><br>**Wave Ic (hospital visit)**<br><br>- Blood pressure (40, 41)<br>- Glucose tolerance test (35)<br>- ECG (42)<br>- Lung function (43)<br>- Urine collection (44)<br>- Lipid profile and clotting factors (45, 46)	912 (90%) of the 1018 selected participants were interviewed at their homes (Wave Ib), 741 (81%) agreed to visit the AMC clinic (Wave Ic). There were no differences in the mean birth weight of the 741 who attended the clinic and the 2414 children in the original cohort.

For peer review only

**Wave II**<br><br>(Age 58)<br><br>2002-2004	**Wave IIa (hospital visit)**<br><br>- Questionnaires (General information, medical information, lifestyle factors, physical activity, weight history reproductive history, transgenerational effects, self-perceived health) (47-54)<br>- Glucose tolerance test (55-57)<br>- Anthropometrics<br>- Blood pressure<br>- ECG (58)<br>- Urine collection (44)<br>- Cognitive function (59)<br>- Ultrasound examinations of the arterial walls of the carotid and femoral arteries (60, 61)<br>- Physical function (62)<br>- Psychological Stress tests (63-66)<br>- Genomic DNA from blood plasma (67-71)<br><br>**Wave IIb (hospital visit)**<br><br>- Intravenous glucose tolerance test in a subsample (*n*=94) (59)<br><br>**Wave IIc (hospital visit)**<br><br>- Synacthen test in a subsample (*n*=98) (69)	860 (60%) of 1423 eligible candidates agreed to participate. There were no differences in mean birth weight between responders and non-responders.

For peer review only

**Wave III**<br><br>(Age 63)<br><br>2008 - 2009	- Questionnaires (General information, medical information, lifestyle factors, physical activity, weight history reproductive history, transgenerational effects, self-perceived health) (72-74)<br>- Transgenerational effects based on F2 questionnaire (General information, birth characteristics, self-perceived health, exercise, medical information, lifestyle factors) (75)<br>- F0-F1-F2 (grandmother-parent-child) buccal swab for DNA methylation	601 (44%) of 1372 eligible candidates agreed to participate. 483 F2s (grandchildren) were willing to participate, of which 360 (74.5%) completed the questionnaire. Birthweight or gestational age did not differ between F1 participants and F1 non-participants.
**Wave IV**<br><br>(age 68)<br><br>2013	- Questionnaires (General information, medical information, lifestyle factors, physical activity, weight history reproductive history, transgenerational effects, self-perceived health)<br>- Anthropometrics<br>- Blood pressure<br>- Glucose concentration (non-fasting)<br>- Lipid profile (non-fasting)<br>- Cognitive function<br>- Brain Imaging (MRI) (white matter hyper intensities, cerebral micro bleeds, total cortical, hippocampal and lacunar volume, brain perfusion, resting brain state conditions, BrainAge, brain perfusion) (76-78)<br>- Ageing parameters (Stroke incidence, fractures and osteoporosis, physical performance, visual acuity, cataract, hearing) (79)<br>- Cellular aging (telomere length) (80)	Equal samples were drawn randomly from each of the groups (1307 eligible candidates in total) until the number of 50 people per exposure group (born before famine, exposed in early gestation, conceived after famine) agreeing to participate was reached. A total number of 151 participants of an eligible group of 268 cohort members (56%) were visited at home. There was a difference in birth weight between the participants (*n*=150) and the nonparticipants (*n*=1,157) of the eligible group (*n*=1,307), 3,438 vs 3,346g; *p* = .03.
**Wave V**	- Questionnaires (including self-perceived health, daily life functioning, pain complaints, mood, memory, attention, and cognition, diseases, tasks and activities	595 (49%) of 1207 eligible candidates agreed to participate. Data analyses are currently ongoing.

For peer review only

(age 73)<br><br>2018	in daily life, social activities, quality of life, medical care consumption, stressful events, health problems resulting from stressful events, and childhood experiences)	
Wave VI-(Ongoing)<br><br>(age 74-75)<br><br>2019 - 2020	- Questionnaires (General information, medical information, lifestyle factors, physical activity, weight history reproductive history, transgenerational effects, self-perceived health)<br>- Anthropometrics<br>- Glucose (non-fasting)<br>- Lipid profile (non-fasting)<br>- Blood pressure<br>- Cognitive function<br>- Brain Imaging (MRI) (white matter hyper intensities, cerebral micro bleeds, structural total and area brain volumes, brain perfusion, BrainAge, resting brain state conditions, active brain state conditions during Stroop selective attention task)	*Ongoing*

For peer review only

Findings to date

1. Prenatal famine exposure has lasting effects on health

Table 3 gives an overview of findings in the Dutch famine birth cohort study. Exposure to famine was associated with a three-fold higher risk for (42) and earlier onset of coronary heart disease (CHD) (58), a more atherogenic lipid profile (45), glucose intolerance (35, 55), a disturbed blood coagulation (46), microalbuminuria and a decrease in creatinine clearance (44), as well as more respiratory complaints and obstructive airways disease in adulthood (43). Also, those exposed to famine during gestation were more likely to consume a high-fat diet (48), and more often perceived their own health as 'poor' (37). Although there was no indication that prenatal undernutrition increased resting blood pressure (40), those exposed to undernutrition *in utero* had a higher increase in systolic blood pressure in response to a stressful assignment compared to unexposed individuals (63). Moreover, they performed worse on a selective attention task, a cognitive ability that usually declines with increasing age (59). Women prenatally exposed to famine were more centrally obese (39), had a five times increased risk for developing breast cancer (52), and an overall higher mortality rate, as well as a higher cardiovascular mortality, cancer mortality, and breast cancer mortality (81). In men, exposure to famine during gestation was associated with smaller intracranial volume (76). Moreover, their brain appeared older, as assessed by a machine-learning pattern recognition method that is able to estimate individual brain ages based on T1-weighted MR images, and overall perfusion of the brain was worse compared to controls (77, 78). Also, prenatal exposure to famine was associated with increased symptoms of depression and anxiety (49), and a lower physical performance score (79) in men. In terms of transgenerational effects, babies born to women exposed to famine prenatally were more adipose as neonates, and parents that had been exposed to famine in utero themselves more often reported their child to have a poorer health

(54). Offspring of men exposed to famine in utero had an increased weight and a higher BMI at age 37 years (75).

2. The effects were dependent of its timing during gestation (and the organs and tissues growing at that time)

Our findings suggest that famine exposure during periods of rapid organ growth permanently affects organ structure. The majority of adverse effects of prenatal famine on several (precursors of) chronic diseases were observed specifically in those exposed during **early** gestation (37, 39, 42, 45, 46, 48, 49, 52, 58, 59, 63, 76-79, 81). Since organogenesis takes place in early gestation it is not surprising that exposure to undernutrition during this critical period of development has the most detrimental effects on later life physical and mental health. The effects on renal function and respiratory complaints were observed only in those who had experienced famine in utero during *mid* gestation (43, 44). This fits with the stage of rapid expansion of nephron number and of rapid bronchial tree branching in mid-pregnancy in fetuses (82). Finally, across all trimesters, but particularly in *mid and late* gestation, famine was associated with offspring glucose intolerance in later life (35, 55). During mid and late gestation, the endocrine pancreas undergoes rapid proliferation (83). Altogether, our findings are highly suggestive of specific windows of increased sensitivity for negative effects of famine on various organ systems, depending on the gestational period in which cell division is at its peak.

3. The effects were independent of size at birth

One of the findings of the Dutch famine birth cohort study is that babies born after exposure to the famine in ***mid*** (difference = -165 gr, 95%CI -220 to -109) or ***late*** (difference = -257 gr, 95%CI = -312 to -202) gestation had lower birth weights, whereas babies born after exposure to the famine in ***early*** (difference = 79 gr, 95%CI = 16 to 143) gestation had higher birth weight

compared to controls (36) (Table 1). This is tremendously striking, since the long-term programming effects of prenatal maternal undernutrition on adult health have traditionally been assumed to be mediated by poor fetal growth and hence marked by a low birth weight. However, we found that the majority of the effects of prenatal famine exposure on adverse health outcomes in adults were observed in those exposed to famine in ***early*** gestation, and thus, in those with normal birth weight. Moreover, additional adjustment for birthweight showed that the effect of prenatal exposure to famine on the adult physical and mental health outcomes were independent of birthweight. In other words, prenatal famine had a major negative affect on adult health, without affecting birth weight. Our findings thus suggest that adverse effects of maternal famine exposure on fetal organ development and function are independent of size at birth.

Further information, including a list of publications can be found on the cohort's web-page [www.hongerwinter.nl].

only

Table 3. Adult characteristics of the Dutch famine birth cohort study from the age of 50 years, divided in columns based upon the gestational period of prenatal famine exposure.

Age	Ref	Adult characteristics	Born before	Late gestation	Mid gestation	Early gestation	Conceived after	Mean (sd)	*n*
**50**	(42)	Coronary Heart Disease (%)	3.8	2.5	0.9	**8.8***	2.6	3.3	736
	(45)	LDL:HDL cholesterol[1]	2.91	2.82	2.69	3.26	2.94	2.90 (1.53)	704
	(35)	120 min. Glucose[1] (mmol/L)	5.7	**6.3***	**6.1***	**6.1***	5.9	6.0 (1.4)	702
		120 min. insulin[1] (mmol/L)	160	**200***	**190***	**207***	181	181 (2.4)	694
	(46)	Fibrinogen (g/l)	3.02	3.05	3.05	3.21	3.10	3.07 (0.6)	725
		Factor VII[1] (% of standard)	127.7	130.7	132.7	**116.9***	132.6	129.4 (1.4)	725
	(40)	SBP (mmHg)	126.0	127.4	124.8	123.4	125.1	125.5 (15.5)	739
		DBP (mmHg)	86.2	86.4	84.4	84.8	85.2	85.6 (9.9)	739
	(39)	BMI[1] (kg/m^2)	26.7	26.7	26.6	**28.1***♀	27.2	27.0 (1.2)	741
		Waist circumference (cm)	91.8	92.4	91.0	**95.6***♀	92.5	92.3 (13)	741
	(37)	Perceived health poor (%)	4.5	6.4	3.7	**10.3***	5.3	5.5	912
	(44)	Microalbuminuria (ACR ≥ 2.5) (%)	8	7	**12***	9	4	7	724
	(43)	FEV1/FVC (x100)	0.72	0.72	0.72	0.72	0.74	0.72 (0.1)	726

For peer review only

		Obstructive airways disease (%)	15.5	15.0	**24.8***	23.0	17.3	18.1	726
		Total IgE[1] (IU/ml)	31.0	27.2	28.9	27.9	33.1	30.4 (4.6)	726
	(84)	Mortality 18–50 years (%)	5.3	3.9	2.8	5.0	3.6	-	2254
**Age**	**Ref**	**Adult characteristics**	**Born before**	**Late gestation**	**Mid gestation**	**Early gestation**	**Conceived after**	**Mean (sd)**	***n***
**58**	(63)	ΔSBP (mmHg) Stroop Response	19	20	19	**23***	19	20 (16)	721
	(55, 56)	120 min. Glucose[1] (mmol/L)	5.8	**6.2***	**6.2***	**6.2***	5.9	6.0	678
		120 min. insulin[1] (pmol/L)	242	**263***	**254***	**269***	240	249 (2.1)	672
	(48)	>39% of energy from fat (%)	25.9	28.7	20.2	**40.7***	24.9	-	730
	(59)	Stroop Task Score[2] (% correct)	42.3	36.5	40.0	**27.5***	43.9	38.5 (55.7)	714
		AH4 test Score[2] (% correct)	70.9	72.4	71.8	76.0	73.3	72.4 (19.0)	727
		Memory task Retrieval (%)	81.8	78.5	83.2	79.7	79.6	80.7(19.9)	583
		Mirror Task Errors per round[2]	13	12	10	14	10	12 (30)	643
	(52)	Breast Cancer (%)	2.8	3.7	3.9	**8.7***	0.8	3.2	475
	(85)	Mortality 18-57 years (%)	9.1	4.4	6.0	6.5	5.9	-	2245

For peer review only

	(49)	HADS-A score	4.5	4.7	5.1	5.8	5.1	4.9 (3.1)	639
		HADS-A ≥ 8 points (OR)	1.0 (ref)	0.7	1.1	**2.7***♂	1.0	-	369
		HADS-D	3.1	3.0	3.1	**4.9***♂	3.6	3.2 (3.1)	369
		HADS-D ≥ 8 points (OR)	1.0 (ref)	0.9	0.6	1.7	1.0	-	369
**Age**	**Ref**	**Adult characteristics**	**Born before**	**Late gestation**	**Mid gestation**	**Early gestation**	**Conceived after**	**Mean (sd)**	***n***
**63**	(81)	Overall adult mortality (HR)	1.0 (ref)	0.8	0.5	**1.9***♀	1.0 (ref)	-	1125
		Cardiovascular mortality (HR)	1.0 (ref)	- (zero deaths)	0.6	**4.6***♀	1.0 (ref)	-	1125
		Cancer mortality (HR)	1.0 (ref)	1.0	0.5	**2.3***♀	1.0 (ref)	-	1125
		Breast cancer mortality (HR)	1.0 (ref)	2.1	1.5	**8.3***♀	1.0 (ref)	-	1125
**Age**	**Ref**	**Adult characteristics**	**Born before**	**Late gestation**	**Mid gestation**	**Early gestation**	**Conceived after**	**Mean (sd)**	***n***
**68**	(76-78)	ICV (ml)	1138	-	-	**1101*** ♂	1176	1138 (85)	52
		BrainAGE score (years)	-1.81	-	-	**2.53***♂	0.53	-	52
		Spatial Coefficient of Variance of a CBF image	0.60	-	-	**0.64***♂	0.59	0.61 (0.08)	50
	(79)	Grip strength (kg)	46.3[3]	-	-	**42.1***	46.3[3]	-	67
		Physical performance score (points)	9.7[3]	-	-	**8.9***	9.7[3]	-	67
**Transgenerational - F2 offspring of F1♀**									

Age	Ref	Adult characteristics	Born before	Exposure to famine	Conceived after	Mean (sd)	*n*
**33**	**(54)**	Birth weight (grams)	3476	3484	3468	-	856
		Ponderal index (kg/m^3)	26.6	**27.8***	26.5	-	856
		F2 poor health[a] (%)	16	18	14	-	856
**Age**	**Ref**	**Adult characteristics**	**Unexposed to famine**	**Exposed to famine**		**Mean (sd)**	***n***
**35**	**(75)**	Adult weight (kg)	78.9	79.1		79.0	209
		Adult BMI[1] (kg/m^2)	25.7	25.0		25.3	209
**Transgenerational - F2 offspring of F1♂**							
**Age**	**Ref**	**Adult characteristics**	**Born before**	**Exposure to famine**	**Conceived after**	**Mean (sd)**	***n***
**30**	**(54)**	Birth weight (grams)	3304	3298	856	**-**	640
		Ponderal index (kg/m^3)	25.9	25.9	26.3	**-**	640
		F2 poor health[a] (%)	12	12	8	**-**	640
**Age**	**Ref**	**Adult characteristics**	**Unexposed to famine**	**Exposed to famine**		**Mean (sd)**	***n***
**35**	**(75)**	Adult weight (kg)	73.5	**78.8***		75.3	151
		Adult BMI[1] (kg/m^2)	23.8	**25.2***		24.3	151

LDL:HDL = Low Density Lipoprotein : High Density Lipoprotein, SBP = Systolic Blood Pressure, DBP = Diastolic Blood Pressure, BMI = Body Mass Index, FEV1/FVC = Forced Expiratory Volume / Forced Vital Capacity, HADS-A = Hospital Anxiety and Depression Scale - Anxiety subscale, HADS-D = Hospital Anxiety and Depression Scale - Depression subscale, ICV = Intracranial Volume, TBV = Total Brain Volume, CBF = Cerebral Blood Flow

[1]: Geometric means (SD)

[2]: Medians (IQR)

[a]: Congenital: asphyxia, developmental delay, Down syndrome, congenital heart disorders; cardiovascular and metabolic: diabetes, acquired cardiovascular conditions, obesity; psychiatric: schizophrenia, depression, suicide (attempt), drug/alcohol dependency; other: accidental, acquired neurological, autoimmune, respiratory, infectious, neoplastic, dermatological conditions.

*: $p <= 0.05$ compared to unexposed (born before and conceived after the famine)

♀ Measured both in men and women, results shown here only include sex-specific significant associations (women)

♂ Measured both in men and women, results shown here only include sex-specific significant associations (men)

For peer review only

Strengths and limitations

A strength of the Dutch famine birth cohort study is its quasi-experimental design. This design reduces the chance that associations between prenatal famine exposure and adult health outcomes are due to genetic inheritance of mother and child or socio-economic status, since exposure to famine was completely independent of maternal characteristics such as age or parity, socio-economic status and other environmental circumstances that may affect long-term health of their children. Nevertheless, exposed and unexposed individuals may have differed on other genetically inherited characteristics which we did not measure. The Dutch hunger winter families study is a different cohort study that used an approach with subtle differences to that of the Dutch famine birth cohort. This cohort was set up in 2003 to study adult health in individuals born in one of three institutions in famine-exposed cities (Amsterdam, Rotterdam, Leiden) to women who had been exposed to famine during or immediately preceding pregnancy. The Dutch hunger winter families study differed from the Dutch famine birth cohort study in one essential aspect: Controls were unexposed same-sex siblings and not unrelated time controls born before or conceived after the famine, to account for any potential genetic influences on later life health outcomes (86).

Another strength of our study design was that, due to the relatively short period of the famine, the exposed individuals are unlikely to substantially differ from unexposed individuals in terms of general socio-environmental conditions, indicating that famine exposure is one of the single remaining factors that can explain differences between both groups. Factors which we cannot control for are, firstly, the possible heightened levels of psychological and/or physical stress in women experiencing famine, which may have affected fetal development also, or, secondly, specific dietary changes that co-occurred at the time of the famine which may have impacted fetal development. Examples of the latter could include tulip bulb consumption.

A relative weakness of the cohort is possible selective conception and survival. During the famine, fertility rates decreased by approximately 50%, due to increased amenorrhea among women (18, 19). Also, perinatal mortality was higher during the famine (19, 87). Both the decreased conception rates and increase in perinatal mortality during the famine may have led to selective births of 'stronger' individuals. However, when we compared maternal characteristics such as socio-economic status, age and parity between those who conceived during the famine and those who gave birth before or conceived after the famine, we found no major differences except that early exposed women were more frequently unmarried. An increase in infant mortality of those with poorer health during the famine is also not likely to explain our findings, as the majority of the associations between prenatal famine and adverse health outcomes was observed in those conceived during the famine.

Another weakness of the study especially as the cohort grows older, is that more resilient individuals may remain alive and in good health, in particular those who are motivated to participate in the study. Selective attrition may have particularly affected the group of exposed individuals, as we have shown that this group was particularly affected by famine exposure in utero, reflected by more deaths (in women) and poorer health outcomes. This asymmetric loss of adults with poorer health may have led to an under estimation of the true effects of prenatal famine on adult health outcomes.

For peer review only

Collaboration

The Dutch famine birth cohort study welcomes opportunities for collaboration; Enquiries should be directed to the Principal Investigator [t.j.roseboom@amsterdamumc.nl].

For peer review only

Acknowledgements

We would like to thank the members of the Dutch famine birth cohort for their participation in our studies. We would also like to thank all researchers and other staff who have worked with us on study design, data collection, assessments, analyses and drafting of manuscript.

Funding and competing interests

The Dutch famine birth cohort study has been funded by the Diabetes Fonds (Netherlands), the Netherlands Heart Foundation, The European Science Foundation (EUROSTRESS), the European Commission (Brainage, Dynahealth, Longitools), Wellbeing (UK), the Medical Research Council (UK), the Dutch Research Council (NWO Aspasia) and the Academic Medical Centre (Amsterdam, The Netherlands). We declare no conflict of interest.

Data sharing statement

Data may be shared under a collaboration agreement. Please contact the Principal Investigator [t.j.roseboom@amsterdaumumc.nl] if you are interested in collaboration.

Author Contributions

ACJR, RCP, SRdR and TJR contributed to parts of the development of methods and parts of the data collection. All authors were involved in data analysis and interpretation. LSB, SRdR and TJR drafted the work. All authors have critically revised this article and approved the final version to be published.

References

1. Law CL, Shiell AW. Is blood pressure inversely related to birth weight? The strength of evidence from a systematic review of the literature. *J Hypertens.* 1996;14:935-41.
2. Davies AA, Smith GD, Ben-Shlomo Y, Litchfield P. Low birth weight is associated with higher adult total cholesterol concentration in men: findings from an occupational cohort of 25,843 employees. *Circulation.* 2004;110(10):1258-62.
3. Whincup PH, Kaye SJ, Owen CG, Huxley R, Cook DG, Anazawa S, et al. Birth weight and risk of type 2 diabetes: a systematic review. *JAMA*. 2008;300(24):2886-97.
4. Knop MR, Geng TT, Gorny AW, Ding R, Li C, Ley SH, et al. Birth Weight and Risk of Type 2 Diabetes Mellitus, Cardiovascular Disease, and Hypertension in Adults: A Meta-Analysis of 7 646 267 Participants From 135 Studies. *J Am Heart Ass.* 2018;7(23):e008870.
5. Osmond C, Barker DJ, Winter PD, Fall CH, Simmonds SJ. Early growth and death from cardiovascular disease in women. *BMJ*. 1993;307(6918):1519-24.
6. Barker DJP, Osmond C, Golding J, Kuh D, Wadsworth MEJ. Growth in utero, bloodpressure in childhood and adult life, and mortality from cardiovascular disease. *Br J Obstet Gynaecol.* 1989;298:564-7.
7. Risnes KR, Vatten LJ, Baker JL, Jameson K, Sovio U, Kajantie E, et al. Birthweight and mortality in adulthood: a systematic review and meta-analysis. *Int J Epidemiol.* 2011;40(3):647-61.
8. Barker DJP, Osmond C. Infant mortality, childhood nutrition and ischeamic heart disease in England and Wales. *Lancet.* 1986;10 May:1077-81.
9. Langley-Evans SC, Gardner DS, Welham SJ. Intrauterine programming of cardiovascular disease by maternal nutritional status. *Nutrition* . 1998;14(1):39-47.
10. Veenendaal MVE, Thangaratinam S, Yates D, Painter RC, de Rooij SR, van der Post JAM, et al. Is the Fetal Origins Hypothesis of Diabetes Supported by Animal Research? A Systematic Review and Meta-Analysis of the Evidence. . *J Diabetes Metab.* 2013; 4:307.
11. Barker DJ. The origins of the developmental origins theory. *J Intern Med*. 2007;261(5):412-7.
12. Barker DJ, Gluckman PD, Godfrey KM, Harding JE, Owens JA, Robinson JS. Fetal nutrition and cardiovascular disease in adult life. *Lancet*. 1993;341(8850):938-41.
13. Nardozza LM, Caetano AC, Zamarian AC, Mazzola JB, Silva CP, Marcal VM, et al. Fetal growth restriction: current knowledge. *Arch Gynecol Obstet.* 2017;295(5):1061-77.
14. Dols MJ, Van Arcken DJ. Food supply and nutrition in the Netherlands during and immediately after World War II. *The Milbank Memorial Fund quarterly.* 1946;24(04):319-58.
15. Rijksarchief sG. Rijksbureau voor de Voedselvoorziening in Oorlogstijd. Verloop der rantsoeneering van de gedistribueerde levensmiddelen voor verschillende leeftijdsgroepen periode 27 april 1941 - 1 October 1944. 1944.
16. Rijksarchief sG. Rijksbureau voor de Voedselvoorziening in Oorlogstijd. Overzichten per dag in de periode 1 October 1944 - 22 juli 1945 voor West-Nederland 1945.
17. Stare FJ. Nutritional conditions in Holland. 1945;3(8):225-7.
18. Burger GCE, Sandstead HR, Drummond JC. Malnutrition and Starvation in Western Netherlands, September 1944 to July 1945. Part I and II. In: Office THGSP, editor. 1948.
19. Stein Z, Susser M, Saenger G, Marolla F. Famine and human development: The Dutch hunger winter of 1944-1945. New York, NY, US: Oxford University Press; 1975.
20. Smith CA. Effects of maternal under nutrition upon the newborn infant in Holland (1944-1945). *J Pediatr.* 1947;30(3):229-43.
21. Sindram IS. De invloed van ondervoeding op de groei van de vrucht. *Ned Tijdschrift Verloskunde Gyneacologie.* 1953;53:30-48.
22. Stein Z, Susser M, Saenger G, Marolla F. Nutrition and mental performance. *Science.* 1972;178(4062):708-13.
23. Ravelli GP, Stein ZA, Susser MW. Obesity in young men after famine exposure in utero and early infancy. *N Engl J Med*. 1976;295(7):349-53.
24. Stein Z, Susser M. The Dutch famine, 1944-1945, and the reproductive process. I. Effects on six indices at birth. *Pediatr Res.* 1975;9(2):70-6.

For peer review only - http://bmjopen.bmj.com/site/about/guidelines.xhtml

25. Stein Z, Susser M. The Dutch famine, 1944-1945, and the reproductive process. II. Interrelations of caloric rations and six indices at birth. *Pediatr Res.* 1975;9(2):76-83.
26. Susser ES, Lin SP. Schizophrenia after prenatal exposure to the Dutch Hunger Winter of 1944-1945. *Arch Gen Psychiatry*. 1992;49(12):983-8.
27. Susser E, Neugebauer R, Hoek HW, Brown AS, Lin S, Labovitz D, et al. Schizophrenia after prenatal famine. Further evidence. *Arch Gen Psychiatry* 1996;53(1):25-31.
28. Brown AS, van Os J, Driessens C, Hoek HW, Susser ES. Further evidence of relation between prenatal famine and major affective disorder. *Am J Psychiatry.* 2000;157(2):190-5.
29. Brown AS, Susser ES, Lin SP, Neugebauer R, Gorman JM. Increased risk of affective disorders in males after second trimester prenatal exposure to the Dutch hunger winter of 1944-45. *Br J Psychiatry*. 1995;166(5):601-6.
30. Hoek HW, Brown AS, Susser E. The Dutch famine and schizophrenia spectrum disorders. *Soc Psychiatry Ppsychiatr Epidemiol*. 1998;33(8):373-9.
31. Hoek HW, Susser E, Buck KA, Lumey LH, Lin SP, Gorman JM. Schizoid personality disorder after prenatal exposure to famine. *Am J Psychiatry.* 1996;153(12):1637-9.
32. Neugebauer R, Hoek HW, Susser E. Prenatal exposure to wartime famine and development of antisocial personality disorder in early adulthood. *JAMA*. 1999;282(5):455-62.
33. Stein AD, Lumey LH. The relationship between maternal and offspring birth weights after maternal prenatal famine exposure: the Dutch Famine Birth Cohort Study. *Hum Biol*. 2000;72(4):641-54.
34. Lumey LH, Ravelli AC, Wiessing LG, Koppe JG, Treffers PE, Stein ZA. The Dutch famine birth cohort study: design, validation of exposure, and selected characteristics of subjects after 43 years follow-up. *Paediatr Perinat Epidemiol*. 1993;7(4):354-67.
35. Ravelli AC, van der Meulen JH, Michels RP, Osmond C, Barker DJ, Hales CN, et al. Glucose tolerance in adults after prenatal exposure to famine. *Lancet*. 1998;351(9097):173-7.
36. Ravelli AC. Prenatal exposure to the Dutch famine and glucose tolerance and obesity at age 50: (AMC-UvA); 1999.
37. Roseboom TJ, Van Der Meulen JH, Ravelli AC, Osmond C, Barker DJ, Bleker OP. Perceived health of adults after prenatal exposure to the Dutch famine. *Paediatr Perinat Epidemiol*. 2003;17(4):391-7.
38. Painter RC, Westendorp RGJ, de Rooij SR, Osmond C, Barker DJP, Roseboom TJ. Increased reproductive success of women after prenatal undernutrition. *Hum Reprod*. 2008;23(11):2591-5.
39. Ravelli AC, van Der Meulen JH, Osmond C, Barker DJ, Bleker OP. Obesity at the age of 50 y in men and women exposed to famine prenatally. *Am J Clin Nutr*. 1999;70(5):811-6.
40. Roseboom TJ, van der Meulen JH, Ravelli AC, van Montfrans GA, Osmond C, Barker DJ, et al. Blood pressure in adults after prenatal exposure to famine. *J Hypertens*. 1999;17(3):325-30.
41. Roseboom TJ, van der Meulen JH, van Montfrans GA, Ravelli AC, Osmond C, Barker DJ, et al. Maternal nutrition during gestation and blood pressure in later life. *J Hypertens*. 2001;19(1):29-34.
42. Roseboom TJ, van der Meulen JH, Osmond C, Barker DJ, Ravelli AC, Schroeder-Tanka JM, et al. Coronary heart disease after prenatal exposure to the Dutch famine, 1944-45. *Heart*. 2000;84(6):595-8.
43. Lopuhaä CE, Roseboom TJ, Osmond C, Barker DJ, Ravelli AC, Bleker OP, et al. Atopy, lung function, and obstructive airways disease after prenatal exposure to famine. *Thorax.* 2000;55(7):555-61.
44. Painter RC, Roseboom TJ, van Montfrans GA, Bossuyt PM, Krediet RT, Osmond C, et al. Microalbuminuria in adults after prenatal exposure to the Dutch famine. *J Am Soc Nephrol*. 2005;16(1):189-94.
45. Roseboom TJ, van der Meulen JH, Osmond C, Barker DJ, Ravelli AC, Bleker OP. Plasma lipid profiles in adults after prenatal exposure to the Dutch famine. *Am J Clin Nutr*. 2000;72(5):1101-6.
46. Roseboom TJ, van der Meulen JH, Ravelli AC, Osmond C, Barker DJ, Bleker OP. Plasma fibrinogen and factor VII concentrations in adults after prenatal exposure to famine. *Br J Hhaematol.* 2000;111(1):112-7.
47. Horenblas J, de Rooij SR, Roseboom TJ. The risk of stroke after prenatal exposure to famine. *J Dev Orig Health Dis*. 2017;8(6):658-64.

For peer review only

48. Lussana F, Painter RC, Ocke MC, Buller HR, Bossuyt PM, Roseboom TJ. Prenatal exposure to the Dutch famine is associated with a preference for fatty foods and a more atherogenic lipid profile. *Am J Clin Nutr*. 2008;88(6):1648-52.
49. de Rooij SR, Painter RC, Phillips DI, Raikkonen K, Schene AH, Roseboom TJ. Self-reported depression and anxiety after prenatal famine exposure: mediation by cardio-metabolic pathology? *J Dev Orig Health Dis.* 2011;2(3):136-43.
50. de Rooij SR, Painter RC, Swaab DF, Roseboom TJ. Sexual orientation and gender identity after prenatal exposure to the Dutch famine. *Arch Sex Behav*. 2009;38(3):411-6.
51. Klooker TK, Braak B, Painter RC, de Rooij SR, van Elburg RM, van den Wijngaard RM, et al. Exposure to severe wartime conditions in early life is associated with an increased risk of irritable bowel syndrome: a population-based cohort study. *Am J Gastroenterol*. 2009;104(9):2250-6.
52. Painter RC, De Rooij SR, Bossuyt PM, Osmond C, Barker DJ, Bleker OP, et al. A possible link between prenatal exposure to famine and breast cancer: a preliminary study. *Am J Human Biol.* 2006;18(6):853-6.
53. van Abeelen AFM, de Rooij SR, Osmond C, Painter RC, Veenendaal MVE, Bossuyt PMM, et al. The sex-specific effects of famine on the association between placental size and later hypertension. *Placenta.* 2011;32(9):694-8.
54. Painter RC, Osmond C, Gluckman P, Hanson M, Phillips DI, Roseboom TJ. Transgenerational effects of prenatal exposure to the Dutch famine on neonatal adiposity and health in later life. *BJOG.* 2008;115(10):1243-9.
55. de Rooij SR, Painter RC, Roseboom TJ, Phillips DI, Osmond C, Barker DJ, et al. Glucose tolerance at age 58 and the decline of glucose tolerance in comparison with age 50 in people prenatally exposed to the Dutch famine. *Diabetologia*. 2006;49(4):637-43.
56. de Rooij SR, Painter RC, Phillips DI, Osmond C, Michels RP, Godsland IF, et al. Impaired insulin secretion after prenatal exposure to the Dutch famine. *Diabetes Care*. 2006;29(8):1897-901.
57. de Rooij SR, Painter RC, Holleman F, Bossuyt PM, Roseboom TJ. The metabolic syndrome in adults prenatally exposed to the Dutch famine. *Am J Clin Nutr*. 2007;86(4):1219-24.
58. Painter RC, de Rooij SR, Bossuyt PM, Simmers TA, Osmond C, Barker DJ, et al. Early onset of coronary artery disease after prenatal exposure to the Dutch famine. *Am J Clin Nutr* 2006;84(2):322-7; quiz 466-7.
59. de Rooij SR, Wouters H, Yonker JE, Painter RC, Roseboom TJ. Prenatal undernutrition and cognitive function in late adulthood. *PNAS.* 2010;107(39):16881-6.
60. Painter RC, de Rooij SR, Hutten BA, Bossuyt PM, de Groot E, Osmond C, et al. Reduced intima media thickness in adults after prenatal exposure to the Dutch famine. *Atherosclerosis.* 2007;193(2):421-7.
61. Painter RC, de Rooij SR, Bossuyt PM, de Groot E, Stok WJ, Osmond C, et al. Maternal nutrition during gestation and carotid arterial compliance in the adult offspring: the Dutch famine birth cohort. *J Hypertens.* 2007;25(3):533-40.
62. Veenendaal MVE, de Rooij SR, Painter RC, Robinson S, Osmond C, Sayer AA, et al. Grip strength at age 58 after prenatal exposure to the Dutch famine. *J Aging Res Clin Pract.* 2012;1.
63. Painter RC, de Rooij SR, Bossuyt PM, Phillips DI, Osmond C, Barker DJ, et al. Blood pressure response to psychological stressors in adults after prenatal exposure to the Dutch famine. *J Hypertens.* 2006;24(9):1771-8.
64. de Rooij SR, Painter RC, Phillips DI, Osmond C, Michels RP, Bossuyt PM, et al. Hypothalamic-pituitary-adrenal axis activity in adults who were prenatally exposed to the Dutch famine. *Eur J Endocrinol*. 2006;155(1):153-60.
65. de Rooij SR, Painter RC, Phillips DI, Osmond C, Tanck MW, Bossuyt PM, et al. Cortisol responses to psychological stress in adults after prenatal exposure to the Dutch famine. *Psychoneuroendocrinology.* 2006;31(10):1257-65.
66. de Rooij SR, Jones A, Phillips DI, Osmond C, Karemaker JM, Roseboom TJ, et al. Prenatal Undernutrition and Autonomic Function in Adulthood. *Psychosom Med*. 2016;78(9):991-7.
67. de Rooij SR, Painter RC, Phillips DI, Osmond C, Tanck MW, Defesche JC, et al. The effects of the Pro12Ala polymorphism of the peroxisome proliferator-activated receptor-gamma2 gene on glucose/insulin metabolism interact with prenatal exposure to famine. *Diabetes Care*. 2006;29(5):1052-7.

For peer review only

68. van Hoek M, Langendonk JG, de Rooij SR, Sijbrands EJ, Roseboom TJ. Genetic variant in the IGF2BP2 gene may interact with fetal malnutrition to affect glucose metabolism. *Diabetes*. 2009;58(6):1440-4.
69. Botden IP, Zillikens MC, de Rooij SR, Langendonk JG, Danser AH, Sijbrands EJ, et al. Variants in the SIRT1 gene may affect diabetes risk in interaction with prenatal exposure to famine. *Diabetes Care*. 2012;35(2):424-6.
70. Veenendaal MV, Costello PM, Lillycrop KA, de Rooij SR, van der Post JA, Bossuyt PM, et al. Prenatal famine exposure, health in later life and promoter methylation of four candidate genes. *J Dev Orig Health Dis*. 2012;3(6):450-7.
71. Lussana F, de Rooij SR, Veenendaal M, Razzari C, Fontana G, Faioni EM, et al. Prevalence of factor V Leiden and G20210A prothrombin mutation in the Dutch Famine Birth Cohort: a possible survival advantage? *Thromb Haemost*. 2012;108(2):399-401.
72. Carroll D, Ginty AT, Painter RC, Roseboom TJ, Phillips AC, de Rooij SR. Systolic blood pressure reactions to acute stress are associated with future hypertension status in the Dutch Famine Birth Cohort Study. *Int J Psychophys*. 2012;85(2):270-3.
73. Ginty AT, Carroll D, Roseboom TJ, Phillips AC, de Rooij SR. Depression and anxiety are associated with a diagnosis of hypertension 5 years later in a cohort of late middle-aged men and women. *J Hum Hypertens*. 2013;27(3):187-90.
74. de Rooij SR, Veenendaal MV, Raikkonen K, Roseboom TJ. Personality and stress appraisal in adults prenatally exposed to the Dutch famine. *Early Hum Dev*. 2012;88(5):321-5.
75. Veenendaal MV, Painter RC, de Rooij SR, Bossuyt PM, van der Post JA, Gluckman PD, et al. Transgenerational effects of prenatal exposure to the 1944-45 Dutch famine. *BJOG*. 2013;120(5):548-53.
76. de Rooij SR, Caan MW, Swaab DF, Nederveen AJ, Majoie CB, Schwab M, et al. Prenatal famine exposure has sex-specific effects on brain size. *Brain*. 2016;139(Pt 8):2136-42.
77. Franke K, Gaser C, Roseboom TJ, Schwab M, de Rooij SR. Premature brain aging in humans exposed to maternal nutrient restriction during early gestation. *NeuroImage*. 2018;173:460-71.
78. de Rooij SR, Mutsaerts H, Petr J, Asllani I, Caan MWA, Groot P, et al. Late-life brain perfusion after prenatal famine exposure. *Neurobiol Aging*. 2019;82:1-9.
79. Bleker LS, de Rooij SR, Painter RC, van der Velde N, Roseboom TJ. Prenatal Undernutrition and Physical Function and Frailty at the Age of 68 Years: The Dutch Famine Birth Cohort Study. *J Gerontol*. 2016;71(10):1306-14.
80. de Rooij SR, van Pelt AM, Ozanne SE, Korver CM, van Daalen SK, Painter RC, et al. Prenatal undernutrition and leukocyte telomere length in late adulthood: the Dutch famine birth cohort study. *Am J Clin Nutr*. 2015;102(3):655-60.
81. van Abeelen AF, Veenendaal MV, Painter RC, de Rooij SR, Dijkgraaf MG, Bossuyt PM, et al. Survival effects of prenatal famine exposure. *Am J Clin Nutr*. 2012;95(1):179-83.
82. Gasser B, Mauss Y, Ghnassia JP, Favre R, Kohler M, Yu O, et al. A quantitative study of normal nephrogenesis in the human fetus: its implication in the natural history of kidney changes due to low obstructive uropathies. *Fetal Diagn Ther*. 1993;8(6):371-84.
83. Stefan Y, Grasso S, Perrelet A, Orci L. A quantitative immunofluorescent study of the endocrine cell populations in the developing human pancreas. *Diabetes*. 1983;32(4):293-301.
84. Roseboom TJ, van der Meulen JH, Osmond C, Barker DJ, Ravelli AC, Bleker OP. Adult survival after prenatal exposure to the Dutch famine 1944--45. *Paediatr Perinat Epidemiol*. 2001;15(3):220-5.
85. Painter RC, Roseboom TJ, Bossuyt PM, Osmond C, Barker DJ, Bleker OP. Adult mortality at age 57 after prenatal exposure to the Dutch famine. *Eur J Epidemiol*. 2005;20(8):673-6.
86. Lumey LH, Susser ES, Stein AD, Kahn HS, van der Pal-de Bruin KM, Blauw GJ, et al. Cohort Profile: The Dutch Hunger Winter Families Study. *Int J Epidemiol*. 2007;36(6):1196-204.
87. Susser M, Stein Z. Timing in prenatal nutrition: a reprise of the Dutch Famine Study. *Nutr Rev*. 1994;52(3):84-94.

For peer review only

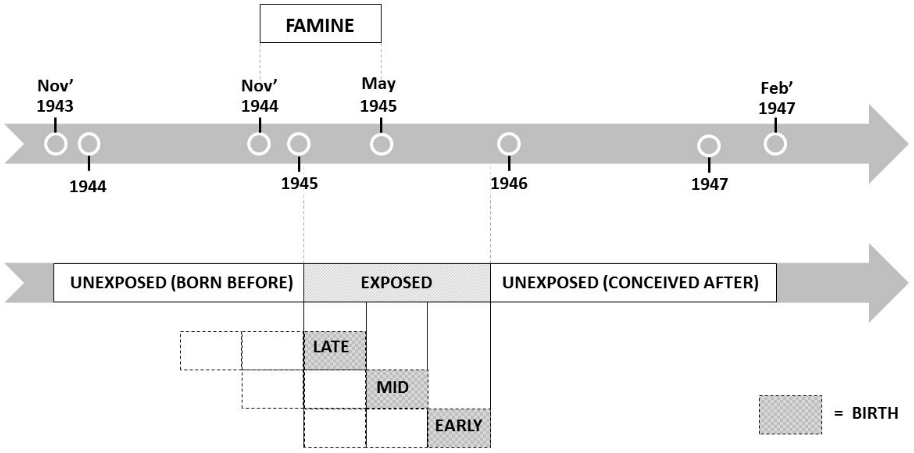

Figure 1. Dutch famine birth cohort schematic presentation of gestational famine exposure.

338x190mm (96 x 96 DPI)

Cohort Profile: The Dutch famine birth cohort (DFBC), a prospective birth cohort study in the Netherlands

Journal:	*BMJ Open*
Manuscript ID	bmjopen-2020-042078.R1
Article Type:	Cohort profile
Date Submitted by the Author:	21-Dec-2020

Complete List of Authors:	Bleker, Laura ; Amsterdam UMC Location AMC, Epidemiology and Data Science<br>de Rooij, Susanne; Amsterdam UMC Location AMC, Epidemiology and Data Science<br>Painter, Rebecca; Amsterdam UMC Location AMC, Obstetrics & Gynaecology<br>Ravelli, Anita C.J.; Amsterdam UMC Location AMC, Medical Informatics<br>Roseboom, Tessa; Amsterdam UMC Location AMC, Clinical Epidemiology and Biostatistics; Obstetrics and Gynaecology

**Primary Subject Heading**:	Epidemiology
Secondary Subject Heading:	Nutrition and metabolism, Obstetrics and gynaecology, Public health
Keywords:	EPIDEMIOLOGY, NUTRITION & DIETETICS, PUBLIC HEALTH

SCHOLARONE™ Manuscripts

I, the Submitting Author has the right to grant and does grant on behalf of all authors of the Work (as defined in the below author licence), an exclusive licence and/or a non-exclusive licence for contributions from authors who are: i) UK Crown employees; ii) where BMJ has agreed a CC-BY licence shall apply, and/or iii) in accordance with the terms applicable for US Federal Government officers or employees acting as part of their official duties; on a worldwide, perpetual, irrevocable, royalty-free basis to BMJ Publishing Group Ltd ("BMJ") its licensees and where the relevant Journal is co-owned by BMJ to the co-owners of the Journal, to publish the Work in this journal and any other BMJ products and to exploit all rights, as set out in our licence.

The Submitting Author accepts and understands that any supply made under these terms is made by BMJ to the Submitting Author unless you are acting as an employee on behalf of your employer or a postgraduate student of an affiliated institution which is paying any applicable article publishing charge ("APC") for Open Access articles. Where the Submitting Author wishes to make the Work available on an Open Access basis (and intends to pay the relevant APC), the terms of reuse of such Open Access shall be governed by a Creative Commons licence – details of these licences and which Creative Commons licence will apply to this Work are set out in our licence referred to above.

Other than as permitted in any relevant BMJ Author's Self Archiving Policies, I confirm this Work has not been accepted for publication elsewhere, is not being considered for publication elsewhere and does not duplicate material already published. I confirm all authors consent to publication of this Work and authorise the granting of this licence.

For peer review only

Cohort Profile: The Dutch famine birth cohort (DFBC), a prospective birth cohort study in the Netherlands

Laura S. Bleker[1], Susanne R. de Rooij[1*], Rebecca C. Painter[2], Anita C.J. Ravelli[2-3], Tessa J. Roseboom[1-2]

[1]Department of Epidemiology and Data Science, Amsterdam UMC, University of Amsterdam, The Netherlands

[2]Department of Obstetrics and Gynecology, Amsterdam UMC, University of Amsterdam, The Netherlands

[3]Department of Medical Informatics, Amsterdam UMC, University of Amsterdam, The Netherlands

*Corresponding author:

Dept. Epidemiology and Data Science, Amsterdam UMC, University of Amsterdam

Meibergdreef 9, 1105 AZ Amsterdam, The Netherlands

Email: s. r.derooij@amsterdamumc.nl

Tel nr: **31 20 56 64660

Word count: 4442

only

For peer review only - http://bmjopen.bmj.com/site/about/guidelines.xhtml

Abstract

Purpose: The Dutch famine birth cohort study was set up to investigate the effects of acute maternal undernutrition of the 1944-1945 Dutch famine during specific stages of gestation on later health, with a particular focus on chronic cardiovascular and metabolic diseases, ageing and mental health.

Participants: The Dutch famine birth cohort consists of 2414 singletons born alive and at term in the Wilhelmina Gasthuis in Amsterdam around the time of the Dutch famine (1943-1947) whose birth records have been kept. The cohort has been traced and studied since 1994, when the first data collection started. The cohort has been interviewed and physically examined in several waves of data collection since that time, allowing repeated measures of a wide range of phenotypic information as well as the collection of biological samples (blood, urine, buccal swabs), functional testing (of heart, lungs, kidney, HPA axis) and imaging of the brain (MRI) and vasculature (ultrasound). Additionally, genetic and epigenetic information was collected. Through linkage with registries, mortality and morbidity information of the entire cohort has been obtained.

Findings to date: Prenatal famine exposure had lasting consequences for health in later life. The effects of famine depended on its timing during gestation and the organs and tissues developing at that time, with most effects after exposure to famine in early gestation. The effects of famine were widespread and affected the structure and function of many organs and tissues, resulted in altered behaviour and increased risks of chronic degenerative diseases, and increased mortality. The effects of famine were independent of size at birth, which suggests that programming may occur without altering size at birth.

Future plans: As the cohort ages, we will be assessing the effects of prenatal undernutrition on (brain) ageing, cognitive decline and dementia, as well as overall morbidity and mortality.

For peer review only

For peer review only - http://bmjopen.bmj.com/site/about/guidelines.xhtml

Strengths and Limitations:

- The quasi-experimental design of the study provides a unique opportunity to investigate effects of undernutrition in pregnancy that would not be ethically possible to study otherwise
- The long period of follow up allows for unusually long term consequences to be investigated decades after the exposure of interest.
- A limitation is the relatively small size of the cohort limiting the statistical power to investigate effects of prenatal famine exposure on outcomes that are rare.
- Selective survival and participation may have introduced bias in the studies which included physical examinations or measurements, but linkage to registries give insight into the degree of selection that has occurred.
- No information is available on the time period between discharge from the hospital after birth and age 50 when the cohort was first traced, leaving limitations for adjusting for potential confounders that may have had an influence during this period.

Registration: The Dutch famine birth cohort is not linked to a clinical trial.

Keywords : Dutch famine, undernutrition, pregnancy, fetal programming, cardiovascular disease, metabolic disease, brain, ageing

For peer review only

For peer review only - http://bmjopen.bmj.com/site/about/guidelines.xhtml

Introduction

Study rationale

The Dutch famine birth cohort study is a historical birth cohort of men and women born around the time of the 1944-1945 Dutch famine in the Wilhelmina Gasthuis in Amsterdam, the Netherlands. The study was set up to investigate the effects of prenatal exposure to the Dutch famine on adult health in order to semi-experimentally test the developmental origins of adult disease hypothesis in a human setting. Based on this hypothesis we expected increased rates of chronic diseases such as type 2 diabetes and cardiovascular disease among those who had been exposed to the Dutch famine prenatally.

This paper describes the rationale for the Dutch famine birth cohort study, why it was set up, how it was set up, and which unique features of the Dutch famine provided the opportunity to semi-experimentally investigate effects of undernutrition in a human setting and allow for the long term consequences to be investigated decades later. We provide an overview of the studies that have been done in the cohort so far, the findings of 25 years of research in this cohort and which insights they provided as well as the implications these insights might have.

The developmental origins of health and disease

Epidemiological studies in populations across the globe have consistently shown that small size at birth is associated with increased risks of chronic degenerative diseases and their biological risk factors such as hypertension, type 2 diabetes and cardiovascular disease .[1-6] The initial observations led to the hypothesis that undernutrition during critical periods of gestation would lead to alterations in the structure and function of organs and tissues developing at the time, resulting in reduced size at birth as well as an increased risk of disease in later life.[7,8]. In experimental studies in animals, undernutrition during pregnancy induced cardiovascular and metabolic changes in the

adult offspring leading to increased rates of disease and reduced lifespan .[9-11] Experimentally investigating the long term effects of undernutrition during gestation in humans would not be ethical nor practically feasible.

The unique setting of the Dutch famine to semi-experimentally investigate effects of undernutrition in humans

The tragic circumstances of the Dutch famine of 1944–1945 created a unique opportunity to assess the effects of prenatal undernutrition on human health in later life. During World War II, part of the Dutch population was exposed to famine during the severe 'hunger winter' of 1944-1945. This historical tragedy has provided the unique opportunity to study short and long-term effects of famine exposure during intrauterine development. The Dutch famine can be viewed as a 'natural experiment', because of its sudden onset and end in a previously well-nourished population. The famine was acute, relatively short, and had an impact on the entire population within the affected regions, minimizing confounding by genetic inheritance or other simultaneously occurring risk factors, such as socio-economic status. Also, throughout the famine, food rations were precisely registered.

Historical events leading to the Dutch famine

In 1944-1945, the Dutch famine hit the western parts of the Netherlands towards the end of World War II, only months before its liberation from the German occupier by the Allied forces. Before the war, nutritional standards of the Dutch population were generally adequate.[12] On the 10th of May 1940, the Netherlands were invaded by German forces, with immediate impact on the Dutch food supply. Food imports from other countries, including the Dutch colonies, were no longer possible, and part of the food produced in the Netherlands was sent to Germany.[12] The National Bureau for Food Distribution had already implemented food rationing and distribution prior to the invasion, and was fully operative throughout the entire country by the end of April 1941. The food rations were

For peer review only - http://bmjopen.bmj.com/site/about/guidelines.xhtml

determined weekly and recorded in detail. However, daily rations remained calorically and nutritionally adequate until September 1944, when they suddenly dropped below 1600 calories.[13,14] On the 17th of September 1944, the Dutch exiled government requested a railroad strike to support the advance of the Allied forces, which by that time had already liberated the southern part of the Netherlands. The German occupier responded by an abrupt ban of food transports to the western part of the Netherlands. By November 1944, food stocks in the large cities in the west of the Netherlands had been exhausted, and the ban was partly lifted to allow food transport across water. However, due to an early and exceptionally harsh winter, waterways and canals had frozen over. Food stocks could no longer be replenished and famine set in for the inhabitants of the large cities in the west, including Amsterdam, Rotterdam and The Hague. The official daily rations per person dropped to below 1000 calories in November 1944 and varied between 400 and 800 calories in the following six months, a period that would later be referred to as the "Hunger Winter". Children aged 1 year or younger were relatively protected, as their official daily ration never dropped below 1000 calories. Although this initially also applied to pregnant and lactating women, this was no longer possible as the food scarcity persisted. On the fifth of May 1945, the western parts of the Netherlands were liberated by The Allied Forces. Within a week, daily rations had rapidly risen above 1000 calories, through supplements by airdrops and the Special Red Cross Feeding Team.[15] By June 1945, rations had risen above 2000 calories. Within weeks, the pre-famine nutritional standards were re-established, and were maintained until the food ration was permanently lifted, 5 years after the war in 1950.[16,17]

Studies of the Dutch famine

Soon after the war ended, researchers realised the unique setting the Dutch famine had unintentionally created to semi-experimentally investigate effects of famine exposure in pregnancy on offspring's health. Since, several research groups have investigated the long-term consequences of prenatal exposure to the Dutch famine. These studies have used a variety of different designs;

For peer review only - http://bmjopen.bmj.com/site/about/guidelines.xhtml

register studies have linked date and place of birth to various outcomes, data from recruitment tests of military conscripts have shown links between prenatal famine exposure (based on time and place of birth) and IQ, mental health and BMI, and birth records have been used to set up cohorts comparing those exposed to famine prenatally with their unexposed same-sex siblings, or to those born in the same hospital either before the famine or conceived after it. Some of the earliest studies on the Dutch famine were published within a few years after World War II by Smith and Sindram, and showed that babies born during the famine were lighter at birth .[18,19]

In the 70's, data from 19-year old Dutch military conscripts was used to investigate effects of prenatal famine exposure on Intelligence Quotient [17] and BMI, showing no effects on intelligence but an increased prevalence of obesity among military conscripts who were exposed to famine during early gestation.[20,21]

Using the Dutch Psychiatry Registry, increased rates of schizophrenia,[22,23, and affective disorders [24,25] were observed in those prenatally exposed to famine. Refined analyses of the military conscripts data, later confirmed these findings and showed higher rates for schizophrenia [26], a schizoid personality [27] and antisocial personality disorder,[28] in men who had been exposed to the Dutch famine prenatally.

Barkers findings of links between small size at birth and later cardiovascular and metabolic disease and the hypothesis that fetal undernutrition would underlie this association [29] led to a collaborative effort between the Academic Medical Center in Amsterdam (the successor of the Wilhelmina Gasthuis that had kept birth records of babies born around the time of the famine) and the Medical Research Council (MRC) Environmental Epidemiology Unit in Southampton, UK, led by Barker, to set up the Dutch famine birth cohort study.

For peer review only - http://bmjopen.bmj.com/site/about/guidelines.xhtml

Cohort description

Eligibility

In 1994, the Dutch famine birth cohort was set up in the Academic Medical Centre in Amsterdam, the Netherlands. All men and women born as term singletons between November 1, 1943 and February 28, 1947 in the Wilhelmina Gasthuis (WG) in Amsterdam were eligible candidates to be included in the Dutch famine birth cohort. The WG was the main maternity hospital in Amsterdam at the time of the famine and birth records were still kept at the city archive of Amsterdam.[30] Only live born singletons after a full-term pregnancy were included (≥259 days gestational age, calculated either from the date of the last menstrual period or by the obstetrician's estimation at first prenatal visit and at physical examination of the child at birth) in order to exclude the potential confounding effects of prematurity or twinning on later health.

Study participants

The birth ledgers, which contained general information on date of birth, name of the parents and identification number were retrieved from the Obstetrics department of the Academic Medical Center (the former WG) in Amsterdam of individuals born between November 1, 1943 and February 28, 1947. From the 5425 records, all individuals exposed to famine, according to our definition (see below), were considered eligible exposed candidates. A random sample of eligible individuals unexposed to famine during gestation (born before the famine or conceived after the famine) was taken. Twins and stillbirths were excluded. This resulted in 2680 eligible candidates for which the detailed medical records were searched and coded in all detail in the city archive of Amsterdam. Of these 2680 individuals, 27 individuals (1.0%) were excluded because their medical records were missing or incomplete, and 239 individuals (8.9%) were excluded based on their preterm birth (<259 days gestational age). Ultimately, 2414 people were considered eligible (821 live born term

For peer review only - http://bmjopen.bmj.com/site/about/guidelines.xhtml

singletons exposed to famine prenatally, 764 live born term singletons born before the famine and 829 live born term singletons conceived after the famine).

Exposure to famine

Exposure to famine during gestation was defined as an average maternal daily ration of less than 1000 calories during any 13-week period of gestation.[31] According to the official daily rations of the general population, children born in Amsterdam between January 7, 1945 and December 8, 1945, were considered to be exposed to famine during prenatal life. Three 16-week periods were distinguished; children who were mainly exposed during ***late*** gestation (born between January 7 and April 28, 1945), ***mid*** gestation (born between April 29 and August 18, 1945) or ***early*** gestation (born between August 19 and December 8, 1945). A sample of the individuals born within one year before the famine or conceived up to one year after the end of the famine were eligible for controls as comparisons. Being unexposed to famine was defined as being born before the famine (born between November 1, 1943 and January 7, 1945), or conceived after the famine (born between December 9, 1945 and February 28, 1947) (Figure 1).

For peer review o

Maternal and birth characteristics

Medical records contained information on maternal characteristics including maternal weight and blood pressure throughout pregnancy as well as a detailed description of delivery and the postpartum period.

Maternal characteristics included maternal age, marital status, reproductive and medical history, and occupation of the head of the family. Blood pressure and maternal weight were measured at every

For peer review only - http://bmjopen.bmj.com/site/about/guidelines.xhtml

prenatal visit. Pelvic measurements were taken, including the interspinous distance (the distance between the anterior superior iliac spines). Maternal weight gain in the third trimester was estimated as the difference between the woman's weight as close as possible to the start of the third trimester and her weight at the last prenatal visit (always within 2 weeks of birth), multiplied by the ratio of trimester duration (13 weeks) and the time interval between the two weight measurements.

Birth characteristics included date of birth, sex, birth weight, and birth length (crown-to-heel). Head circumference was calculated by: π * (biparietal diameter + occipitofrontal diameter) * 0.5. Ponderal index was calculated by: birth weight / (crown-to-heel^2). Placental area was calculated by: π * placental length * placental width * 0.25. At discharge, infant weight and type of feeding (exclusive breastfeeding/exclusive bottle-feeding/a combination) were recorded. Besides information from the medical records for each women the profession of the father / the head of the family was collected using the hospital admission "crib cards" and coded as manual or non-manual work. Table 1 shows the maternal and birth characteristics of the cohort according to the timing of exposure to famine.

For peer review only

Follow-up

In 1994, current addresses of the children born before, during and after the famine were requested from the population registry (Bevolkingsregister) in Amsterdam, which traced 2155 of the 2414 eligible participants. Of these, 265 persons had died, 199 had emigrated, and another 164 refused to share their address, resulting in 1527 (63.3%) of 2155 cases. Power calculations for a study sample to be invited for interviewing and clinical measurements were based on the 120-min glucose concentrations after a 75 gram oral glucose load as the main outcome variable, based on pooled data from three previous MRC Southampton studies which had established a link between birth weight and glucose tolerance in adulthood (Hertfordshire, Preston and Sheffield).[32] To detect an increase

For peer review only - http://bmjopen.bmj.com/site/about/guidelines.xhtml

of 10% in the 120-minute glucose concentration, a sample size of 700 men and women (450 prenatally exposed and 250 unexposed persons) was needed for 93% power and a 5% type 1 error probability (two-sided). A 70% participation rate for the oral glucose load test was assumed and based on this, interviews with 1000 men and women at their homes were planned. We invited all people in each of the 5 exposure/non-exposure groups, starting with those who lived in, or close to, Amsterdam (n=1018) to participate in the first study, of whom 912 (90%) agreed to be interviewed at their homes and 741 (81%) of the interviewed participants visited the clinic for measurements.[31]

Data collection of the Dutch famine birth cohort study has occurred in six study waves from 1994/6 until 2020, with a seventh wave planned in 2021 (See Figure 2 for an overview of study waves). Measurements consisted of a home interview at age 50 and a hospital visit around the ages of 50 (wave I, 1994 - 1996), 58 (wave II, 2002-2004), 63 (wave III, 2008-2009), 68 (wave IV, 2012-2013), and 74 (wave VI, 2019-2020) and completion of a questionnaire by the participants at age 73 (wave V, 2018). . At wave V in 2018, 1207 of the 1527 participants were still living in the Netherlands with known addresses (79%), of which 595 agreed to participate (49%). The fourth and sixth waves occurred in subsamples of the cohort. Ethical approvals for the study have been provided by the Ethics Committee of the Academic Medical Center Amsterdam and have been registered under the numbers: MEC 01 / 215, MEC 08 / 26, MEC 2011/ 305, W17_025 and MEC 2019 / 017.

Measurements

Table 2 gives a summary of the study waves, including the mean ages of the famine exposed participants during that wave, with outcome measures and a description of participation rates. Outcome measures were compared between those exposed to famine in early, mid or late gestation to those prenatally unexposed to famine, mostly by applying regression models with exposures as dummy variables.

For peer review only - http://bmjopen.bmj.com/site/about/guidelines.xhtml

Patient and Public Involvement statement

Cohort members are informed about study aims and outcomes via newsletters and the studies website www.hongerwinter.nl. Cohort members have not been involved in setting up research questions and data collection, study design, recruitment strategies, assessment of study burden and dissemination of study results.

For peer review only

For peer review only - http://bmjopen.bmj.com/site/about/guidelines.xhtml

Table 1. Maternal characteristics and birth outcomes of 2414 singleton children born alive and at term in the Wilhelmina Gasthuis in Amsterdam between 1 November 1943 and 28 February 1947.

		Exposure to Famine				Total (SD)	Missing (*n*)
	Born before n=764	Late gestation n=307	Mid gestation n=297	Early gestation n=217	Conceived after n=829	n=2414	
**Maternal characteristics**							
Age (years)	28.5	30.0	28.1	27.7	28.0	28.3 (6.4)	0
Never married (%)	13.2	9.8	20.2	25.8	16.3	15.8	0
Weight gain third trim. (kg)	3.1	0.0	4.9	5.7	4.2	3.5 (3.2)	732
Weight at last prenatal visit (kg)	66.7	61.8	63.5	67.9	69.1	66.6 (8.7)	281
Interspinous distance (cm)	26.1	25.8	25.5	25.8	26.3	26.0 (1.8)	14
**Birth outcomes**							
	**Born before *n*=764**	**Late gestation *n*=307**	**Mid gestation *n*=297**	**Early gestation *n*=217**	**Conceived after =829**	**Total *n*=2414**	
Boys (%)	53.1	48.2	48.5	49.8	52.5	51.4	
First born (%)	39.7	29.6	36.7	39.2	39.3	37.9	

For peer review only - http://bmjopen.bmj.com/site/about/guidelines.xhtml

Pregnancy duration (days)	285	283	285	287	286	285 (11)	371
Birth weight (g)	3373	3133	3217	3470	3413	3346 (478)	0
Body length (cm)	50.5	49.4	49.8	50.9	50.5	50.3 (2.2)	32
Ponderal index (kg/m^3)	26.1	25.8	26.0	26.2	26.5	26.4 (2.4)	32
Head circumference (cm)	32.9	32.3	32.1	32.8	33.2	32.8 (2.4)	17
Placental diameter (cm)	20.6	19.7	20.0	19.9	20.2	20.2 (2.5)	357

*Adapted from Table 1, Page 59, Ravelli et al. [33]

For peer review only

Table 2. Dutch famine birth cohort study waves, including the mean ages of the participants at examination, the outcome measures and a description of attrition rates.

Phase	Measurement	Loss of follow-up
**Wave I**<br><br>1994-1996	**Wave 1a birth data collection/ exposure definition/ mortality status**	
**Wave I**<br><br>(Age 50)<br><br>1994– 1996	**Wave Ib (home interview)**<br><br>- Questionnaires (General information, medical information, life style factors, physical activity, weight history, reproductive history, EPIC Food frequency questionnaire, self-perceived health and medication use) [34,35]<br>- Anthropometrics [36]<br>- Blood pressure [37,38]<br><br>**Wave Ic (hospital visit)**<br><br>- Blood pressure [37,38]<br>- Glucose tolerance test [31]<br>- ECG [39]<br>- Lung function [40]<br>- Urine collection [41]	912 (90%) of the 1018 selected participants were interviewed at their homes (Wave Ib), 741 (81%) agreed to visit the AMC clinic (Wave Ic). There were no differences in the mean birth weight of the 741 who attended the clinic and the 2414 children in the original cohort.

For peer review only - http://bmjopen.bmj.com/site/about/guidelines.xhtml

	- Lipid profile and clotting factors [42,43]	
**Wave II**<br><br>(Age 58)<br><br>2002-2004	**Wave IIa (hospital visit)**<br><br>- Questionnaires (General information, medical information, lifestyle factors, physical activity, weight history reproductive history, transgenerational effects, self-perceived health and medication use) [44-51]<br>- Glucose tolerance test [52,53]<br>- Anthropometrics<br>- Blood pressure<br>- ECG [54]<br>- Urine collection [41]<br>- Cognitive function [55]<br>- Ultrasound examinations of the arterial walls of the carotid and femoral arteries [56,57]<br>- Physical function [58]<br>- Psychological Stress tests [59-61]<br>- Genomic DNA from blood plasma [62-66]<br><br>**Wave IIb (hospital visit)**	860 (60%) of 1423 eligible candidates agreed to participate. There were no differences in mean birth weight between responders and non-responders.

For peer review only

For peer review only - http://bmjopen.bmj.com/site/about/guidelines.xhtml

	- Intravenous glucose tolerance test in a subsample (*n*=94) [67]<br><br>**Wave IIc (hospital visit)**<br><br>- Synacthen test in a subsample (*n*=98) [68]	
**Wave III**<br><br>(Age 63)<br><br>2008 - 2009	- Questionnaires (General information, medical information, lifestyle factors, physical activity, weight history reproductive history, transgenerational effects, self-perceived health and medication use) [69-71]<br>- Transgenerational effects based on F2 questionnaire (General information, birth characteristics, self-perceived health, exercise, medical information, lifestyle factors) [72]<br>- F0-F1-F2 (grandmother-parent-child) buccal swab for DNA methylation	601 (44%) of 1372 eligible candidates agreed to participate. 483 F2s (grandchildren) were willing to participate, of which 360 (74.5%) completed the questionnaire. Birthweight or gestational age did not differ between F1 participants and F1 non-participants.
**Wave IV**<br><br>(age 68)<br><br>2012-2013	- Questionnaires (General information, medical information, lifestyle factors, physical activity, weight history reproductive history, transgenerational effects, self-perceived health and medication use)<br>- Anthropometrics<br>- Blood pressure<br>- Glucose concentration (non-fasting)<br>- Lipid profile (non-fasting)<br>- Cognitive function	Equal samples were drawn randomly from each of the groups (1307 eligible candidates in total) until the number of 50 people per exposure group (born before famine, exposed in early gestation, conceived after famine) agreeing to participate was reached. A total number of 151 participants of an eligible group of 268 cohort members (56%) were visited at home. There was a difference in

For peer review only - http://bmjopen.bmj.com/site/about/guidelines.xhtml

	- Brain Imaging (MRI) (white matter hyper intensities, cerebral micro bleeds, total cortical, hippocampal and lacunar volume, brain perfusion, resting brain state conditions, BrainAge, brain perfusion) [73-75]<br>- Ageing parameters (Stroke incidence, fractures and osteoporosis, physical performance, visual acuity, cataract, hearing) [76]<br>- Cellular aging (telomere length) [77]	birth weight between the participants (*n*=150) and the nonparticipants (*n*=1,157) of the eligible group (*n*=1,307), 3,438 vs 3,346g; *p* = .03.
**Wave V**<br><br>(age 73)<br><br>2018 - 2019	- Questionnaires (including self-perceived health, daily life functioning, pain complaints, mood, memory, attention, and cognition, diseases, tasks and activities in daily life, social activities, quality of life, medical care consumption, stressful events, health problems resulting from stressful events, and childhood experiences)	595 (49%) of 1207 eligible candidates agreed to participate. Data analyses are currently ongoing.
**Wave VI**<br><br>(age 74)<br><br>2019 - 2020	- Questionnaires (General information, medical information, lifestyle factors, physical activity, weight history reproductive history, transgenerational effects, self-perceived health and medication use)<br>- Anthropometrics<br>- Glucose (non-fasting)<br>- Lipid profile (non-fasting)<br>- Blood pressure<br>- Cognitive function<br>- Brain Imaging (MRI) (white matter hyper intensities, cerebral micro bleeds, structural total and area brain volumes, brain perfusion, BrainAge, resting	Cohort members who underwent MRI scanning in Wave IV were re-invited for this study. A total of 92 participants were seen in this study. Data analyses are currently being set up.

For peer review only

	brain state conditions, active brain state conditions during Stroop selective attention task)	

For peer review only - http://bmjopen.bmj.com/site/about/guidelines.xhtml

Findings to date

Prenatal exposure to undernutrition appears to have (1) permanent effects on human health. The effects of undernutrition (2) depend on its timing during gestation and the organs and systems developing during that critical time window. The effects on later health were most pronounced among those exposed to famine in early gestation. This may not be surprising considering the fact that all organs are formed in early gestation and insufficient food supply during the formation of the organs interferes most with future physiological functions. The effects of famine were independent of size at birth (3) as there were effects of prenatal famine exposure in the absence of effects on body size at birth. This implies that adaptations that enable the foetus to grow can nevertheless have adverse long-term consequences.

Below, we describe the findings according to the 3 main conclusions.

1. ***Prenatal famine exposure has lasting effects on health***

Table 3 gives an overview of findings in the Dutch famine birth cohort study. Exposure to famine was associated with a three-fold higher risk for [39] and earlier onset of coronary heart disease (CHD) ,[54] a more atherogenic lipid profile,[42] glucose intolerance,[31,52) a disturbed blood coagulation,[43] microalbuminuria and a decrease in creatinine clearance,[41] as well as more respiratory complaints and obstructive airways disease in adulthood.[40] Also, those exposed to famine during gestation were more likely to consume a high-fat diet,[45] and more often perceived their own health as 'poor'.[34] Although there was no indication that prenatal undernutrition increased resting blood pressure,[37] those exposed to undernutrition *in utero* had a higher increase in systolic blood pressure in response to a stressful assignment compared to unexposed individuals .[59] Moreover, they performed worse on a selective attention task, a cognitive ability that usually declines with increasing age.[55] Women prenatally exposed to famine were more centrally obese,[36] had a five times increased risk for developing breast cancer,[49] and an overall higher

For peer review only - http://bmjopen.bmj.com/site/about/guidelines.xhtml

mortality rate, as well as a higher cardiovascular mortality, cancer mortality, and breast cancer mortality.[78] In men, exposure to famine during gestation was associated with smaller intracranial volume.[73] Moreover, their brain appeared older, as assessed by a machine-learning pattern recognition method that is able to estimate individual brain ages based on T1-weighted MR images, and overall perfusion of the brain was worse compared to controls.[74,75] Also, prenatal exposure to famine was associated with increased symptoms of depression and anxiety,[46] and a lower physical performance score in men.[76] In terms of transgenerational effects, babies born to women exposed to famine prenatally were more adipose as neonates, and parents that had been exposed to famine in utero themselves more often reported their child to have a poorer health.[51] Offspring of men exposed to famine in utero had an increased weight and a higher BMI at age 37 years.[72]

2. *The effects were dependent of its timing during gestation (and the organs and tissues growing at that time)*

Our findings suggest that famine exposure during periods of rapid organ growth permanently affects organ structure. The majority of adverse effects of prenatal famine on several (precursors of) chronic diseases were observed specifically in those exposed during **early** gestation.[34,36,39,42,43,45,46,49,54,55,59,73-76,78] Since organogenesis takes place in early gestation it is not surprising that exposure to undernutrition during this critical period of development has the most detrimental effects on later life physical and mental health. The effects on renal function and respiratory complaints were observed only in those who had experienced famine in utero during *mid* gestation.[40,41] This fits with the stage of rapid expansion of nephron number and of rapid bronchial tree branching in mid-pregnancy in fetuses.[79,80] Finally, across all trimesters, but particularly in *mid and late* gestation, famine was associated with offspring glucose intolerance in later life.[31,52] During mid and late gestation, the endocrine pancreas undergoes rapid proliferation.[81] Altogether, our findings are highly suggestive of specific windows of

For peer review only - http://bmjopen.bmj.com/site/about/guidelines.xhtml

increased sensitivity for negative effects of famine on various organ systems, depending on the gestational period in which cell division is at its peak.

3. The effects were independent of size at birth

One of the findings of the Dutch famine birth cohort study is that babies born after exposure to the famine in ***mid*** (difference = -165 gr, 95%CI -220 to -109) or ***late*** (difference = -257 gr, 95%CI = -312 to -202) gestation had lower birth weights, whereas babies born after exposure to the famine in ***early*** (difference = 79 gr, 95%CI = 16 to 143) gestation had higher birth weight compared to controls (Table 1).[33] This is tremendously striking, since the long-term programming effects of prenatal maternal undernutrition on adult health have traditionally been assumed to be mediated by poor fetal growth and hence marked by a low birth weight. However, we found that the majority of the effects of prenatal famine exposure on adverse health outcomes in adults were observed in those exposed to famine in ***early*** gestation, and thus, in those with normal birth weight. Moreover, additional adjustment for birthweight showed that the effect of prenatal exposure to famine on the adult physical and mental health outcomes were independent of birthweight. In other words, prenatal famine had a major negative affect on adult health, without affecting birth weight. Our findings thus suggest that adverse effects of maternal famine exposure on fetal organ development and function are independent of size at birth.

Further information, including a list of publications can be found on the cohort's web-page [www.hongerwinter.nl].

For peer review only - http://bmjopen.bmj.com/site/about/guidelines.xhtml

Table 3. Adult characteristics of the Dutch famine birth cohort study from the age of 50 years, divided in columns based upon the gestational period of prenatal famine exposure.

Age	Ref	Adult characteristics	Born before	Late gestation	Mid gestation	Early gestation	Conceived after	Mean (sd)	*n*
**50**	[39]	Coronary Heart Disease (%)	3.8	2.5	0.9	**8.8***	2.6	3.3	736
	[42]	LDL:HDL cholesterol[1]	2.91	2.82	2.69	3.26	2.94	2.90 (1.53)	704
	[31]	120 min. Glucose[1] (mmol/L)	5.7	**6.3***	**6.1***	**6.1***	5.9	6.0 (1.4)	702
		120 min. insulin[1] (mmol/L)	160	**200***	**190***	**207***	181	181 (2.4)	694
	[43]	Fibrinogen (g/l)	3.02	3.05	3.05	3.21	3.10	3.07 (0.6)	725
		Factor VII[1] (% of standard)	127.7	130.7	132.7	**116.9***	132.6	129.4 (1.4)	725
	[37]	SBP (mmHg)	126.0	127.4	124.8	123.4	125.1	125.5 (15.5)	739
		DBP (mmHg)	86.2	86.4	84.4	84.8	85.2	85.6 (9.9)	739
	[36]	BMI[1] (kg/m^2)	26.7	26.7	26.6	**28.1*♀**	27.2	27.0 (1.2)	741
		Waist circumference (cm)	91.8	92.4	91.0	**95.6*♀**	92.5	92.3 (13)	741

For peer review only - http://bmjopen.bmj.com/site/about/guidelines.xhtml

	[34]	Perceived health poor (%)	4.5	6.4	3.7	**10.3***	5.3	5.5	912
	[41]	Microalbuminuria (ACR ≥ 2.5) (%)	8	7	**12***	9	4	7	724
	[40]	FEV1/FVC (x100)	0.72	0.72	0.72	0.72	0.74	0.72 (0.1)	726
		Obstructive airways disease (%)	15.5	15.0	**24.8***	23.0	17.3	18.1	726
		Total IgE[1] (IU/ml)	31.0	27.2	28.9	27.9	33.1	30.4 (4.6)	726
	[82]	Mortality 18–50 years (%)	5.3	3.9	2.8	5.0	3.6	-	2254
**Age**	**Ref**	**Adult characteristics**	**Born before**	**Late gestation**	**Mid gestation**	**Early gestation**	**Conceived after**	**Mean (sd)**	***n***
**58**	[59]	ΔSBP (mmHg) Stroop Response	19	20	19	**23***	19	20 (16)	721
	[52]	120 min. Glucose[1] (mmol/L)	5.8	**6.2***	**6.2***	**6.2***	5.9	6.0	678
		120 min. insulin[1] (pmol/L)	242	**263***	**254***	**269***	240	249 (2.1)	672
	[45]	>39% of energy from	25.9	28.7	20.2	**40.7***	24.9	-	730

For peer review only - http://bmjopen.bmj.com/site/about/guidelines.xhtml

		fat (%)							
	[55]	Stroop Task Score[2] (% correct)	42.3	36.5	40.0	**27.5***	43.9	38.5 (55.7)	714
		AH4 test Score[2] (% correct)	70.9	72.4	71.8	76.0	73.3	72.4 (19.0)	727
		Memory task Retrieval (%)	81.8	78.5	83.2	79.7	79.6	80.7(19.9)	583
		Mirror Task Errors per round[2]	13	12	10	14	10	12 (30)	643
	[49]	Breast Cancer (%)	2.8	3.7	3.9	**8.7***	0.8	3.2	475
	[83]	Mortality 18-57 years (%)	9.1	4.4	6.0	6.5	5.9	-	2245
	[46]	HADS-A score	4.5	4.7	5.1	5.8	5.1	4.9 (3.1)	639
		HADS-A ≥ 8 points (OR)	1.0 (ref)	0.7	1.1	**2.7*♂**	1.0	-	369
		HADS-D	3.1	3.0	3.1	**4.9*♂**	3.6	3.2 (3.1)	369
		HADS-D ≥ 8 points (OR)	1.0 (ref)	0.9	0.6	1.7	1.0	-	369
**Age**	**Ref**	**Adult characteristics**	**Born before**	**Late gestation**	**Mid gestation**	**Early gestation**	**Conceived after**	**Mean (sd)**	***n***
**63**	[78]	Overall adult mortality (HR)	1.0 (ref)	0.8	0.5	**1.9*♀**	1.0 (ref)	-	1125

For peer review only

For peer review only - http://bmjopen.bmj.com/site/about/guidelines.xhtml

		Cardiovascular mortality (HR)	1.0 (ref)	- (zero deaths)	0.6	**4.6*♀**	1.0 (ref)	-	1125
		Cancer mortality (HR)	1.0 (ref)	1.0	0.5	**2.3*♀**	1.0 (ref)	-	1125
		Breast cancer mortality (HR)	1.0 (ref)	2.1	1.5	**8.3*♀**	1.0 (ref)	-	1125
**Age**	**Ref**	**Adult characteristics**	**Born before**	**Late gestation**	**Mid gestation**	**Early gestation**	**Conceived after**	**Mean (sd)**	***n***
**68**	[73-75]	ICV (ml)	1138	-	-	**1101*** ♂	1176	1138 (85)	52
		BrainAGE score (years)	-1.81	-	-	**2.53*** ♂	0.53	-	52
		Spatial Coefficient of Variance of a CBF image	0.60	-	-	**0.64*** ♂	0.59	0.61 (0.08)	50
	[76]	Grip strength (kg)	46.3[3]	-	-	**42.1***	46.3[3]	-	67
		Physical performance score (points)	9.7[3]	-	-	**8.9***	9.7[3]	-	67
**Transgenerational - F2 offspring of F1♀**									
**Age**	**Ref**	**Adult characteristics**	**Born before**	**Exposure to famine**			**Conceived after**	**Mean (sd)**	***n***
	[51]	Birth weight (grams)	3476	3484			3468	-	856

For peer review only - http://bmjopen.bmj.com/site/about/guidelines.xhtml

**33**		Ponderal index (kg/m^3)	26.6	**27.8***	26.5	-	856
		F2 poor health[a] (%)	16	18	14	-	856
**Age**	**Ref**	**Adult characteristics**	**Unexposed to famine**		**Exposed to famine**	**Mean (sd)**	***n***
**35**	[72]	Adult weight (kg)	78.9		79.1	79.0	209
		Adult BMI[1] (kg/m^2)	25.7		25.0	25.3	209
**Transgenerational - F2 offspring of F1♂**							
**Age**	**Ref**	**Adult characteristics**	**Born before**	**Exposure to famine**	**Conceived after**	**Mean (sd)**	***n***
**30**	[51]	Birth weight (grams)	3304	3298	856	-	640
		Ponderal index (kg/m^3)	25.9	25.9	26.3	-	640
		F2 poor health[a] (%)	12	12	8	-	640
**Age**	**Ref**	**Adult characteristics**	**Unexposed to famine**		**Exposed to famine**	**Mean (sd)**	***n***
**35**	[72]	Adult weight (kg)	73.5		**78.8***	75.3	151
		Adult BMI[1] (kg/m^2)	23.8		**25.2***	24.3	151

LDL:HDL = Low Density Lipoprotein : High Density Lipoprotein, SBP = Systolic Blood Pressure, DBP = Diastolic Blood Pressure, BMI = Body Mass Index, FEV1/FVC = Forced Expiratory Volume / Forced

For peer review only - http://bmjopen.bmj.com/site/about/guidelines.xhtml

Vital Capacity, HADS-A = Hospital Anxiety and Depression Scale - Anxiety subscale, HADS-D = Hospital Anxiety and Depression Scale - Depression subscale, ICV = Intracranial Volume, TBV = Total Brain Volume, CBF = Cerebral Blood Flow

[1]: Geometric means (SD)

[2]: Medians (IQR)

[a]: Congenital: asphyxia, developmental delay, Down syndrome, congenital heart disorders; cardiovascular and metabolic: diabetes, acquired cardiovascular conditions, obesity; psychiatric: schizophrenia, depression, suicide (attempt), drug/alcohol dependency; other: accidental, acquired neurological, autoimmune, respiratory, infectious, neoplastic, dermatological conditions.

*: $p <= 0.05$ compared to unexposed (born before and conceived after the famine)

♀ Measured both in men and women, results shown here only include sex-specific significant associations (women)

♂ Measured both in men and women, results shown here only include sex-specific significant associations (men)

For peer review only

Strengths and limitations

A strength of the Dutch famine birth cohort study is its quasi-experimental design. This design reduces the chance that associations between prenatal famine exposure and adult health outcomes are due to genetic inheritance of mother and child or socio-economic status, since exposure to famine was completely independent of maternal characteristics such as age or parity, socio-economic status and other environmental circumstances that may affect long-term health of their children. Nevertheless, exposed and unexposed individuals may have differed on other genetically inherited characteristics which we did not measure. The Dutch hunger winter families study is a different cohort study that used an approach with subtle differences to that of the Dutch famine birth cohort.[84] This cohort was set up in 2003 to study adult health in individuals born in one of three institutions in famine-exposed cities (Amsterdam, Rotterdam, Leiden) to women who had been exposed to famine during or immediately preceding pregnancy. The Dutch hunger winter families study differed from the Dutch famine birth cohort study in one essential aspect: Controls were unexposed same-sex siblings and not unrelated time controls born before or conceived after the famine, to account for any potential genetic influences on later life health outcomes.[84] Interestingly, the findings between the two cohorts are strikingly similar, and suggest that the consequences that both studies find of prenatal famine exposure are due to a causal effect of famine exposure rather than due to confounding.

Another strength of our study design was that, due to the relatively short period of the famine, the exposed individuals are unlikely to substantially differ from unexposed individuals in terms of general socio-environmental conditions, indicating that famine exposure is one of the single remaining factors that can explain differences between both groups. Factors which we cannot control for are, firstly, the possible heightened levels of psychological and/or physical stress in women experiencing famine, which may have affected fetal development also, or, secondly, specific dietary changes that co-occurred at the time of the famine which may have impacted fetal development. Examples of the

For peer review only - http://bmjopen.bmj.com/site/about/guidelines.xhtml

latter could include tulip bulb consumption. Our analyses do not allow us to dissect effects of lack of food from stress or increased rates of infection due to famine exposure. Interestingly, the effects of postnatal famine exposure which has occurred among those born before the famine seem to be limited as the adult health outcomes of those born before the famine and those conceived after it are strikingly similar.

A relative weakness of the cohort is possible selective conception and survival. During the famine, fertility rates decreased by approximately 50%, due to increased amenorrhea among women.[16,17] Also, perinatal mortality was higher during the famine.[17,85] Both the decreased conception rates and increase in perinatal mortality during the famine may have led to selective births of 'stronger' individuals. However, when we compared maternal characteristics such as socio-economic status, age and parity between those who conceived during the famine and those who gave birth before or conceived after the famine, we found no major differences except that early exposed women were more frequently unmarried. An increase in infant mortality of those with poorer health during the famine is also not likely to explain our findings, as the majority of the associations between prenatal famine and adverse health outcomes was observed in those conceived during the famine.

Another weakness of the study especially as the cohort grows older, is that more resilient individuals may remain alive and in good health, in particular those who are motivated to participate in the study. Selective attrition may have particularly affected the group of exposed individuals, as we have shown that this group was particularly affected by famine exposure in utero, reflected by more deaths (in women) and poorer health outcomes. This asymmetric loss of adults with poorer health may have led to an under estimation of the true effects of prenatal famine on adult health outcomes.

A final limitation to the study is that we have no information on the time period between discharge from the hospital after birth and age 50 when the cohort was first traced, making it impossible to adjust for potential confounders that may have had an influence during this period.

For peer review only - http://bmjopen.bmj.com/site/about/guidelines.xhtml

Similar findings in different settings

Studies in other settings, of famines with different durations and severity support these findings and suggest that the results of studies on the Dutch famine are not uniquely linked to the Dutch famine situation, but rather reflect biologically fundamental processes of human plasticity.[86] A study in Nigeria showed that people who had been exposed to the Biafran famine (1967–1970) prenatally had increased rates of hypertension and type 2 diabetes.[87] Similarly, studies of the Great Leap Forward famine in China have shown similar effects of prenatal famine exposure on later risks of diabetes, hypertension and schizophrenia.[88-90] And similarly studies of Austrian [91] and Ukrainian [92] showed increased risks of diabetes among those who had been exposed to famine prenatally.

Implications

Studies of the Dutch and other famines show that those faced with undernutrition during the critical earliest stages of development have increased rates of chronic generative disease in adult life. These findings teach us the fundamental importance of a good start in life. Adequately feeding women before and during pregnancy will allow future generations to reach their full potential and lead healthier and more productive lives, ultimately leading to healthier and more equal future.

Collaboration

The Dutch famine birth cohort study welcomes opportunities for collaboration; Enquiries should be directed to the Principal Investigator [t.j.roseboom@amsterdamumc.nl].

For peer review only - http://bmjopen.bmj.com/site/about/guidelines.xhtml

Acknowledgements

We would like to thank the members of the Dutch famine birth cohort for their participation in our studies. We would also like to thank all researchers and other staff who have worked with us on study design, data collection, assessments, analyses and drafting of manuscript.

Funding and competing interests

The Dutch famine birth cohort study has been funded by the Diabetes Fonds (The Netherlands, Grant Number NA), the Netherlands Heart Foundation (NHS2001B087, NHS2007B083), The European Science Foundation (EUROSTRESS-DOME project), the European Commission (Brainage (Seventh Framework Program Project 279281), Dynahealth (Horizon 2020 Project 633595), Longitools (Horizon 2020 Project 874739), Wellbeing (UK, Grant Number NA), the Medical Research Council (UK, Grant Number NA), the Dutch Research Council (NWO Aspasia Project 015014039) and the Academic Medical Centre (Amsterdam, The Netherlands, Grant number NA). We declare no conflict of interest.

Data sharing statement

Data are available upon reasonable request. Please contact the Principal Investigator [t.j.roseboom@amsterdaumumc.nl] if you are interested in collaboration.

Author Contributions

ACJR, RCP, SRdR and TJR contributed to parts of the development of methods and parts of the data collection. All authors were involved in data analysis and interpretation. LSB, SRdR and TJR drafted the work. All authors have critically revised this article and approved the final version to be published.

Figure 1. Dutch famine birth cohort schematic presentation of gestational famine exposure.

For peer review only - http://bmjopen.bmj.com/site/about/guidelines.xhtml

Figure 2. Dutch famine birth cohort schematic presentation of study waves.

For peer review only

For peer review only - http://bmjopen.bmj.com/site/about/guidelines.xhtml

References

1. Mu M, Wang SF, Sheng J, Zhao Y, Li HZ, Hu CL, et al. Birth weight and subsequent blood pressure: a meta-analysis. Arch Cardiovasc Dis. 2012;105(2):99-113.
2. Davies AA, Smith GD, Ben-Shlomo Y, Litchfield P. Low birth weight is associated with higher adult total cholesterol concentration in men: findings from an occupational cohort of 25,843 employees. Circulation. 2004;110(10):1258-62.
3. Whincup PH, Kaye SJ, Owen CG, Huxley R, Cook DG, Anazawa S, et al. Birth weight and risk of type 2 diabetes: a systematic review. JAMA. 2008;300(24):2886-97.
4. Knop MR, Geng TT, Gorny AW, Ding R, Li C, Ley SH, et al. Birth Weight and Risk of Type 2 Diabetes Mellitus, Cardiovascular Disease, and Hypertension in Adults: A Meta-Analysis of 7 646 267 Participants From 135 Studies. Journal of the American Heart Association. 2018;7(23):e008870.
5. Mohseni R, Mohammed SH, Safabakhsh M, Mohseni F, Monfared ZS, Seyyedi J, et al. Birth Weight and Risk of Cardiovascular Disease Incidence in Adulthood: a Dose-Response Meta-analysis. Curr Atheroscler Rep. 2020;22(3):12.
6. Risnes KR, Vatten LJ, Baker JL, Jameson K, Sovio U, Kajantie E, et al. Birthweight and mortality in adulthood: a systematic review and meta-analysis. Int J Epidemiol. 2011;40(3):647-61.
7. Barker DJP, Osmond C. Infant mortality, childhood nutrition and ischeamic heart disease in England and Wales. Lancet. 1986;10 May:1077-81.
8. Hoffman DJ, Reynolds RM, Hardy DB. Developmental origins of health and disease: current knowledge and potential mechanisms. Nutr Rev. 2017;75(12):951-70.
9. Van Abeelen AF, Veenendaal MV, Painter RC, De Rooij SR, Thangaratinam S, Van Der Post JA, et al. The fetal origins of hypertension: a systematic review and meta-analysis of the evidence from animal experiments of maternal undernutrition. J Hypertens. 2012;30(12):2255-67.
10. Veenendaal MV, Thangaratinam S, Yates D, Painter R, Rooij D, Jam vdP, et al. Is the Fetal Origins Hypothesis of Diabetes Supported by Animal Research? A Systematic Review and Meta-Analysis of the Evidence. Journal of diabetes & metabolism. 2013;2013:1-8.
11. Chavatte-Palmer P, Tarrade A, Rousseau-Ralliard D. Diet before and during Pregnancy and Offspring Health: The Importance of Animal Models and What Can Be Learned from Them. Int J Environ Res Public Health. 2016;13(6).
12. Dols MJL, Arcken DJAM. Food supply and nutrition in the Netherlands during and immediately after world war II. 1st ed. New York: Milbank Memorial Fund; 1946 1946.
13. Oorlogstijd RvdVi. Verloop der rantsoeneering van de gedistribueerde levensmiddelen voor verschillende leeftijdsgroepen periode 27 April 1941 - 1 October 1944. 1944.
14. Oorlogstijd RvdVi. Overzichten per dag periode 1 Oct 1944 - 22 juli 1945 voor West-Nederland. 1945.
15. Stare FJ. Nutitional conditions in Holland. Nutrition Reviews. 1945;3:225-7.
16. Burger GCE, Sandstead HR, Drummond JC. Malnutrition and Starvation in Western Netherlands, September 1944 to July 1945. Part I and II. The Hague: General State Printing Office; 1948 1948.
17. Stein Z, Susser M, Saenger G, Morolla F. Famine and Human Development. The Dutch Hunger Winter of 1944-45. New York: Oxford University Press; 1975 1975.
18. Smith CA. Effects of maternal undernutrition upon the newborn infant in Holland (1944-1945). Journal of Paediatrics. 1947;30:229-43.
19. Sindram IS. De invloed van ondervoeding op de groei van de vrucht. Nederlands Tijdschrift voor Verloskunde en Gynaecologie. 1953;53:30-48.
20. Stein Z, Susser M, Saenger G, Marolla F. Nutrition and mental performance. Science. 1972;178:708-13.
21. Ravelli GP, Stein ZA, Susser MW. Obesity in young men after famine exposure in utero and early infancy. New England Journal of Medicine. 1976;7:349-54.

For peer review only - http://bmjopen.bmj.com/site/about/guidelines.xhtml

22. Susser ES, Lin SP. Schizophrenia after prenatal exposure to the Dutch Hunger Winter of 1944-1945. Arch Gen Psychiatry. 1992;49:983-8.
23. Susser E, Neugebauer R, Hoek HW, Brown AS, Lin S, Labovitz D, et al. Schizophenia after prenatal famine. Arch Gen Psychiatry. 1996;53:25-31.
24. Brown A, Van Os J, Driessens C, Hoek H, Susser E. Further evidence of relation between prenatal famine and major affective disorder. American Journal of Psychiatry. 2000;157(2):190-5.
25. Brown AS, Susser ES, Lin SP, Neugebauer R, Gorman JM. Increased risk of affective disorders in males after second trimester prenatal exposure to the Dutch Hunger Winter of 1944-45. British Journal of Psychiatry. 1995;166:601-6.
26. Hoek HW, Brown AS, Susser E. The Dutch Famine and schizophrenia spectrum disorders. Social Psychiatry and Psychiatric Epidemiology. 1998;33:373-9.
27. Hoek HW, Susser EZ, Buck K, Lumey LH, Lin SP, Gorman JM. Schizoid personality disorder after prenatal exposure to famine. American Journal of Psychiatry. 1996;153:1637-9.
28. Neugebauer R, Hoek HW, Susser E. Prenatal exposure to Wartime famine and development of antisocial personality disorder in early adulthood. JAMA. 1999;282:455-62.
29. Barker DJP. The fetal origins of diseases of old age. European Journal of Clinical Nutrition. 1992;46 suppl 3:S3-S9.
30. Lumey LH, Ravelli ACJ, Wiessing LG, Koppe JG, Treffers PE, Stein ZA. The Dutch famine birth cohort study: design, validation of exposure, and selected characteristics of subjects after 43 years follow-up. Paediatric and Perinatal Epidemiology. 1993;7(4):354-67.
31. Ravelli ACJ, van der Meulen JHP, Michels RPJ, Osmond C, Barker DJP, Hales CN, et al. Glucose tolerance in adults after prenatal exposure to famine. Lancet. 1998;351:173-7.
32. Barker DJP. Fetal nutrition and cardiovascular disease in later life. British Medical Bulletin. 1997;53:96-108.
33. Ravelli AC. Prenatal exposure to the Dutch famine and glucose tolerance and obesity at age 50. Amsterdam: AMC-University of Amsterdam; 1999.
34. Roseboom TJ, Van Der Meulen JH, Ravelli AC, Osmond C, Barker DJ, Bleker OP. Perceived health of adults after prenatal exposure to the Dutch famine. PaediatrPerinatEpidemiol. 2003;17(4):391-7.
35. Painter RC, Westendorp RG, de Rooij SR, Osmond C, Barker DJ, Roseboom TJ. Increased reproductive success of women after prenatal undernutrition. HumReprod. 2008;23(11):2591-5.
36. Ravelli ACJ, van der Meulen JHP, Osmond C, Barker DJP, Bleker OP. Obesity at the age of 50 y in men and women exposed to famine prenatally. AJCN. 1999;70:811-6.
37. Roseboom TJ, van der Meulen JHP, Ravelli ACJ, van Montfrans GA, Osmond C, Barker DJP, et al. Blood pressure in adults after prenatal exposure to famine. J Hypertens. 1999;17:325-30.
38. Roseboom TJ, van der Meulen JHP, van Montfrans GA, Ravelli ACJ, Osmond C, Barker DJP, et al. Maternal nutrition during gestation and blood pressure in later life. J Hypertens. 2001;19:29-34.
39. Roseboom TJ, van der Meulen JHP, Osmond C, Barker DJP, Ravelli ACJ, Tanka JS, et al. Coronary heart disease after prenatal exposure to the Dutch famine, 1944-45. Heart. 2000;84:595-8.
40. Lopuhaa C, Roseboom TJ, Osmond C, Barker DJP, Ravelli ACJ, Bleker OP, et al. Atopy, lung function and obstructive airways disease after prenatal exposure to famine. Thorax. 2000;55:555-61.
41. Painter RC, Roseboom TJ, van Montfrans GA, Bossuyt PM, Krediet RT, Osmond C, et al. Microalbuminuria in adults after prenatal exposure to the Dutch famine. JAmSocNephrol. 2005;16(1):189-94.
42. Roseboom TJ, van der Meulen JHP, Osmond C, Barker DJP, Ravelli ACJ, Bleker OP. Plasma lipid profiles in adults after prenatal exposure to the Dutch famine. AJCN. 2000;72:1101-6.
43. Roseboom TJ, van der Meulen JHP, Ravelli ACJ, Osmond C, Barker DJP, Bleker OP. Plasma fibrinogen and factor VII concentrations in adults after prenatal exposure to famine. Br J Haematol. 2000;111:112-7.
44. Horenblas J, de Rooij SR, Roseboom TJ. The risk of stroke after prenatal exposure to famine. J Dev Orig Health Dis. 2017;8(6):658-64.

For peer review only - http://bmjopen.bmj.com/site/about/guidelines.xhtml

45. Lussana F, Painter RC, Ocke MC, Buller HR, Bossuyt PM, Roseboom TJ. Prenatal exposure to the Dutch famine is associated with a preference for fatty foods and a more atherogenic lipid profile. AmJ ClinNutr. 2008;88(6):1648-52.
46. de Rooij SR, Painter RC, Phillips DI, Raikkonen K, Schene AH, Roseboom TJ. Self-reported depression and anxiety after prenatal famine exposure: mediation by cardio-metabolic pathology? J Dev Orig Health Dis. 2011;2(3):136-43.
47. de Rooij SR, Painter RC, Swaab DF, Roseboom TJ. Sexual orientation and gender identity after prenatal exposure to the Dutch famine. Arch Sex Behav. 2009;38(3):411-6.
48. Klooker TK, Braak B, Painter RC, de Rooij SR, van Elburg RM, van den Wijngaard RM, et al. Exposure to severe wartime conditions in early life is associated with an increased risk of irritable bowel syndrome: a population-based cohort study. Am J Gastroenterol. 2009;104(9):2250-6.
49. Painter RC, de Rooij SR, Bossuyt PM, Osmond C, Barker DJ, Bleker OP, et al. A possible link between prenatal exposure to famine and breast cancer: A preliminary study. AmJHumBiol. 2006;18(6):853-6.
50. van Abeelen AFM, de Rooij SR, Osmond C, Painter RC, Veenendaal MVE, Bossuyt PMM, et al. The sex-specific effects of famine on the association between placental size and later hypertension. Placenta. 2011;32(9):694-8.
51. Painter RC, Osmond C, Gluckman P, Hanson M, Phillips DI, Roseboom TJ. Transgenerational effects of prenatal exposure to the Dutch famine on neonatal adiposity and health in later life. Bjog. 2008;115(10):1243-9.
52. de Rooij SR, Painter RC, Roseboom TJ, Phillips DI, Osmond C, Barker DJ, et al. Glucose tolerance at age 58 and the decline of glucose tolerance in comparison with age 50 in people prenatally exposed to the Dutch famine. Diabetologia. 2006;49:637-43.
53. de Rooij SR, Painter RC, Holleman F, Bossuyt PM, Roseboom TJ. The metabolic syndrome in adults prenatally exposed to the Dutch famine. AmJ ClinNutr. 2007;86(4):1219-24.
54. Painter RC, de Rooij SR, Roseboom TJ, Bossuyt PMM, Simmers TA, Osmond C, et al. Early onset of coronary artery disease after prenatal exposure to the Dutch famine. AJCN. 2006;84(2):322-7.
55. de Rooij SR, Wouters H, Yonker JE, Painter RC, Roseboom TJ. Prenatal undernutrition and cognitive function in late adulthood. ProcNatlAcadSciUSA. 2010;107(39):16881-6.
56. Painter RC, de Rooij SR, Hutten BA, Bossuyt PM, de GE, Osmond C, et al. Reduced intima media thickness in adults after prenatal exposure to the Dutch famine. Atherosclerosis. 2006;193(2):421-7.
57. Painter RC, de Rooij SR, Bossuyt PM, de GE, Stok WJ, Osmond C, et al. Maternal nutrition during gestation and carotid arterial compliance in the adult offspring: the Dutch famine birth cohort. JHypertens. 2007;25(3):533-40.
58. Veenendaal MVEdR, S.R.; Painter R.C.; Robinson, S.; Osmond, C.; Sayer, A.A.; Roseboom, T.J. Grip strength at age 58 after prenatal exposure to the Dutch famine. J Aging Res Clin Pract. 2012;1.
59. Painter RC, de Rooij SR, Bossuyt PM, Phillips DI, Osmond C, Barker DJ, et al. Blood pressure response to psychological stressors in adults after prenatal exposure to the Dutch famine. J Hypertens. 2006;24(9):1771-8.
60. de Rooij SR, R.C. P, Phillips DIW, Osmond C, Tanck MWT, P.M.M. B, et al. Cortisol responses to psychological stress in adults after prenatal exposure to the Dutch famine. Psychoneuroendocrinology. 2006;31(10):1257-65.
61. de Rooij SR, Jones A, Phillips DI, Osmond C, Karemaker JM, Roseboom TJ, et al. Prenatal Undernutrition and Autonomic Function in Adulthood. Psychosom Med. 2016;78(9):991-7.
62. de Rooij SR, Painter RC, Phillips DI, Osmond C, Tanck MW, Defesche JC, et al. The effects of the Pro12Ala polymorphism of the peroxisome proliferator-activated receptor-gamma2 gene on glucose/insulin metabolism interact with prenatal exposure to famine. Diabetes Care. 2006;29(5):1052-7.

For peer review only - http://bmjopen.bmj.com/site/about/guidelines.xhtml

63. van Hoek M, Langendonk JG, de Rooij SR, Sijbrands EJ, Roseboom TJ. Genetic variant in the IGF2BP2 gene may interact with fetal malnutrition to affect glucose metabolism. Diabetes. 2009;58(6):1440-4.
64. Botden IP, Zillikens MC, de Rooij SR, Langendonk JG, Danser AH, Sijbrands EJ, et al. Variants in the SIRT1 gene may affect diabetes risk in interaction with prenatal exposure to famine. Diabetes Care. 2012;35(2):424-6.
65. Veenendaal MV, Costello PM, Lillycrop KA, de Rooij SR, van der Post JA, Bossuyt PM, et al. Prenatal famine exposure, health in later life and promoter methylation of four candidate genes. J Dev Orig Health Dis. 2012;3(6):450-7.
66. Lussana F, de Rooij SR, Veenendaal M, Razzari C, Fontana G, Faioni EM, et al. Prevalence of factor V Leiden and G20210A prothrombin mutation in the Dutch Famine Birth Cohort: a possible survival advantage? Thromb Haemost. 2012;108(2):399-401.
67. de Rooij SR, Painter RC, Phillips DI, Osmond C, Michels RP, Godsland IF, et al. Impaired insulin secretion after prenatal exposure to the dutch famine. Diabetes Care. 2006;29(8):1897-901.
68. de Rooij SR, Painter RC, Phillips DI, Osmond C, Michels RP, Bossuyt PM, et al. Hypothalamic-pituitary-adrenal axis activity in adults who were prenatally exposed to the Dutch famine. EurJEndocrinol. 2006;155(1):153-60.
69. Carroll D, Ginty AT, Painter RC, Roseboom TJ, Phillips AC, de Rooij SR. Systolic blood pressure reactions to acute stress are associated with future hypertension status in the Dutch Famine Birth Cohort Study. International journal of psychophysiology : official journal of the International Organization of Psychophysiology. 2012;85(2):270-3.
70. Ginty AT, Carroll D, Roseboom TJ, Phillips AC, de Rooij SR. Depression and anxiety are associated with a diagnosis of hypertension 5 years later in a cohort of late middle-aged men and women. J Hum Hypertens. 2013;27(3):187-90.
71. de Rooij SR, Veenendaal MV, Raikkonen K, Roseboom TJ. Personality and stress appraisal in adults prenatally exposed to the Dutch famine. Early HumDev. 2011.
72. Veenendaal MV, Painter RC, de Rooij SR, Bossuyt PM, van der Post JA, Gluckman PD, et al. Transgenerational effects of prenatal exposure to the 1944-45 Dutch famine. Bjog. 2013;120(5):548-53.
73. de Rooij SRC, M.W.A.; Schwaab, D.F.; nederveen, A.J.; Majoie, C.B.; Schwab, M.; Painter, R.C.; Roseboom, T.J. Prenatal famine exposure has sex-specific effects on brain size. Brain. 2016;In press.
74. Franke K, Gaser C, Roseboom TJ, Schwab M, de Rooij SR. Premature brain aging in humans exposed to maternal nutrient restriction during early gestation. NeuroImage. 2018;173:460-71.
75. de Rooij SR, Mutsaerts H, Petr J, Asllani I, Caan MWA, Groot P, et al. Late-life brain perfusion after prenatal famine exposure. Neurobiology of aging. 2019;82:1-9.
76. Bleker LS, de Rooij SR, Painter RC, van der Velde N, Roseboom TJ. Prenatal Undernutrition and Physical Function and Frailty at the Age of 68 Years: The Dutch Famine Birth Cohort Study. J Gerontol A Biol Sci Med Sci. 2016;71(10):1306-14.
77. de Rooij SR, van Pelt AM, Ozanne SE, Korver CM, van Daalen SK, Painter RC, et al. Prenatal undernutrition and leukocyte telomere length in late adulthood: the Dutch famine birth cohort study. Am J Clin Nutr. 2015;102(3):655-60.
78. van Abeelen AF, Veenendaal MV, Painter RC, de Rooij SR, Dijkgraaf MG, Bossuyt PM, et al. Survival effects of prenatal famine exposure. AmJClinNutr. 2012;95(1):179-83.
79. Gasser B, Mauss Y, Ghnassia JP, Favre R, Kohler M, Yu O, et al. A quantitative study of normal nephrogenesis in the human fetus: its implication in the natural history of kidney changes due to low obstructive uropathies. Fetal Diagn Ther. 1993;8(6):371-84.
80. Sparrow MP, Weichselbaum M, McCray PB. Development of the innervation and airway smooth muscle in human fetal lung. Am J Respir Cell Mol Biol. 1999;20(4):550-60.
81. Stefan Y, Grasso S, Perrelet A, Orci L. A quantitative immunofluorescent study of the endocrine cell populations in the developing human pancreas. Diabetes. 1983;32(4):293-301.

For peer review only - http://bmjopen.bmj.com/site/about/guidelines.xhtml

82. Roseboom TJ, van der Meulen JHP, Osmond C, Barker DJP, Ravelli ACJ, Bleker OP. Adult survival after prenatal exposure to the Dutch famine 1944-45. Paediatric and Perinatal Epidemiology. 2001;15:220-5.
83. Painter RC, Roseboom TJ, Bossuyt PM, Osmond C, Barker DJ, Bleker OP. Adult mortality at age 57 after prenatal exposure to the Dutch famine. Eur J Epidemiol. 2005;20:673-6.
84. Lumey LH, Stein AD, Kahn HS, van der Pal-de Bruin KM, Blauw GJ, Zybert PA, et al. Cohort profile: the Dutch Hunger Winter families study. Int J Epidemiol. 2007;36(6):1196-204.
85. Susser M, Stein Z. Timing in prenatal nutrition: a reprise of the Dutch Famine Study. Nutrition Reviews. 1994;52:84-94.
86. Gluckman PD, Beedle AS, Hanson MA, Yap EP. Developmental perspectives on individual variation: implications for understanding nutritional needs. Nestle Nutr Workshop Ser Pediatr Program. 2008;62:1-9; disucssion -12.
87. Hult M, Tornhammar P, Ueda P, Chima C, Bonamy AK, Ozumba B, et al. Hypertension, diabetes and overweight: looming legacies of the Biafran famine. PloS one. 2010;5(10):e13582.
88. Meng R, Lv J, Yu C, Guo Y, Bian Z, Yang L, et al. Prenatal famine exposure, adulthood obesity patterns and risk of type 2 diabetes. Int J Epidemiol. 2018;47(2):399-408.
89. Wang PX, Wang JJ, Lei YX, Xiao L, Luo ZC. Impact of fetal and infant exposure to the Chinese Great Famine on the risk of hypertension in adulthood. PloS one. 2012;7(11):e49720.
90. Xu MQ, Sun WS, Liu BX, Feng GY, Yu L, Yang L, et al. Prenatal malnutrition and adult schizophrenia: further evidence from the 1959-1961 Chinese famine. Schizophr Bull. 2009;35(3):568-76.
91. Thurner S, Klimek P, Szell M, Duftschmid G, Endel G, Kautzky-Willer A, et al. Quantification of excess risk for diabetes for those born in times of hunger, in an entire population of a nation, across a century. Proc Natl Acad Sci U S A. 2013;110(12):4703-7.
92. Lumey LH, Khalangot MD, Vaiserman AM. Association between type 2 diabetes and prenatal exposure to the Ukraine famine of 1932-33: a retrospective cohort study. Lancet Diabetes Endocrinol. 2015;3(10):787-94.

For peer review only

For peer review only - http://bmjopen.bmj.com/site/about/guidelines.xhtml

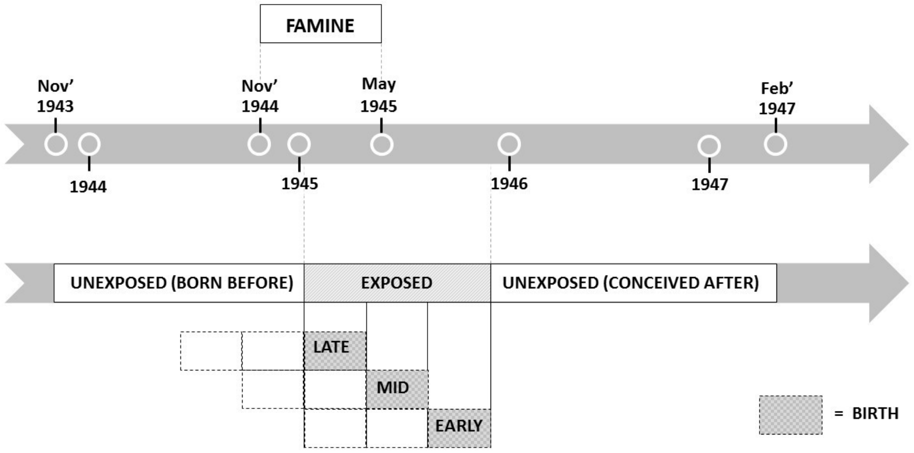

Figure 1. Dutch famine birth cohort schematic presentation of gestational famine exposure.

338x190mm (96 x 96 DPI)

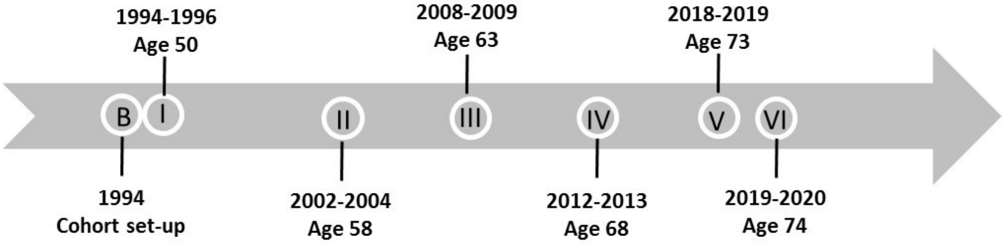

Figure 2. Dutch famine birth cohort schematic presentation of study waves.

214x56mm (120 x 120 DPI)
